# Phylogeny-Aware Analysis of Metagenome Community Ecology Based on Matched Reference Genomes while Bypassing Taxonomy

**DOI:** 10.1128/msystems.00167-22

**Published:** 2022-04-04

**Authors:** Qiyun Zhu, Shi Huang, Antonio Gonzalez, Imran McGrath, Daniel McDonald, Niina Haiminen, George Armstrong, Yoshiki Vázquez-Baeza, Julian Yu, Justin Kuczynski, Gregory D. Sepich-Poore, Austin D. Swafford, Promi Das, Justin P. Shaffer, Franck Lejzerowicz, Pedro Belda-Ferre, Aki S. Havulinna, Guillaume Méric, Teemu Niiranen, Leo Lahti, Veikko Salomaa, Ho-Cheol Kim, Mohit Jain, Michael Inouye, Jack A. Gilbert, Rob Knight

**Affiliations:** a School of Life Sciences, Arizona State University, Tempe, Arizona, USA; b Center for Fundamental and Applied Microbiomics, Arizona State University, Tempe, Arizona, USA; c Department of Pediatrics, School of Medicine, University of California San Diego, La Jolla, California, USA; d Center for Microbiome Innovation, Jacobs School of Engineering, University of California San Diego, La Jolla, California, USA; e Faculty of Dentistry, The University of Hong Kong, Hong Kong SAR, China; f Division of Biological Sciences, University of California San Diego, La Jolla, California, USA; g IBM T. J. Watson Research Center, Yorktown Heights, New York, USA; h Bioinformatics and Systems Biology Program, University of California San Diego, La Jolla, California, USA; i Google, Mountain View, California, USA; j Department of Bioengineering, University of California San Diego, La Jolla, California, USA; k Scripps Institution of Oceanography, University of California San Diego, La Jolla, California, USA; l Department of Public Health and Welfare, Finnish Institute for Health and Welfare, Helsinki, Finland; m Institute for Molecular Medicine Finland, HiLIFE, University of Helsinki, Helsinki, Finland; n Cambridge Baker Systems Genomics Initiative, Baker Heart and Diabetes Institute, Melbourne, Victoria, Australia; o Department of Infectious Diseases, Central Clinical School, Monash University, Melbourne, Victoria, Australia; p Department of Internal Medicine, University of Turku, Turku, Finland; q Division of Medicine, Turku University Hospital, Finland; r Department of Computing, University of Turku, Turku, Finland; s IBM Almaden Research Center, San Jose, California, USA; t Department of Medicine, University of California San Diego, La Jolla, California, USA; u Department of Pharmacology, University of California San Diego, La Jolla, California, USA; v Department of Public Health and Primary Care, Cambridge University, Cambridge, United Kingdom; w Department of Computer Science and Engineering, University of California San Diego, La Jolla, California, USA; Oregon State University

**Keywords:** operational genomic unit, taxonomy independent, reference phylogeny, UniFrac, supervised learning, metagenomics

## Abstract

We introduce the operational genomic unit (OGU) method, a metagenome analysis strategy that directly exploits sequence alignment hits to individual reference genomes as the minimum unit for assessing the diversity of microbial communities and their relevance to environmental factors. This approach is independent of taxonomic classification, granting the possibility of maximal resolution of community composition, and organizes features into an accurate hierarchy using a phylogenomic tree. The outputs are suitable for contemporary analytical protocols for community ecology, differential abundance, and supervised learning while supporting phylogenetic methods, such as UniFrac and phylofactorization, that are seldom applied to shotgun metagenomics despite being prevalent in 16S rRNA gene amplicon studies. As demonstrated in two real-world case studies, the OGU method produces biologically meaningful patterns from microbiome data sets. Such patterns further remain detectable at very low metagenomic sequencing depths. Compared with taxonomic unit-based analyses implemented in currently adopted metagenomics tools, and the analysis of 16S rRNA gene amplicon sequence variants, this method shows superiority in informing biologically relevant insights, including stronger correlation with body environment and host sex on the Human Microbiome Project data set and more accurate prediction of human age by the gut microbiomes of Finnish individuals included in the FINRISK 2002 cohort. We provide Woltka, a bioinformatics tool to implement this method, with full integration with the QIIME 2 package and the Qiita web platform, to facilitate adoption of the OGU method in future metagenomics studies.

**IMPORTANCE** Shotgun metagenomics is a powerful, yet computationally challenging, technique compared to 16S rRNA gene amplicon sequencing for decoding the composition and structure of microbial communities. Current analyses of metagenomic data are primarily based on taxonomic classification, which is limited in feature resolution. To solve these challenges, we introduce operational genomic units (OGUs), which are the individual reference genomes derived from sequence alignment results, without further assigning them taxonomy. The OGU method advances current read-based metagenomics in two dimensions: (i) providing maximal resolution of community composition and (ii) permitting use of phylogeny-aware tools. Our analysis of real-world data sets shows that it is advantageous over currently adopted metagenomic analysis methods and the finest-grained 16S rRNA analysis methods in predicting biological traits. We thus propose the adoption of OGUs as an effective practice in metagenomic studies.

## INTRODUCTION

The rapidly developing field of shotgun metagenomics has enabled higher-resolution determination of microbial community structure, well beyond that of 16S rRNA gene amplicon sequencing. This determination is achieved through algorithms that match DNA sequence data, typically obtained by high-throughput sequencing methods, against a database that contains a comprehensive collection of reference genomes (whole-genome or marker sequences). Taxonomy, the hierarchical classification of organisms (1), is a central part of this process. Many tools (e.g., see references 2–5) have been developed to estimate the abundance of taxonomic groups in the community—a process referred to as taxonomic profiling (6). Recently, cluster analysis has been useful in defining taxonomic groups, both in building reference databases and in matching sequence data to these references. For example, species-level clusters of genomes have been identified by the average nucleotide identity (7, 8), and sequences can be recruited to species clusters based on the phylogenetic distance informed by marker genes (9, 10). Regardless of the analytical method, the end product of these analyses is a per-sample table with features representing taxonomic units at a fixed rank (e.g., species, genus, or above). This table can then be analyzed using statistical approaches such as those implemented in QIIME 2 (11) to explore the structure and diversity of communities and their relevance to biological factors.

Although this taxonomy-centered strategy is effective, it has limitations compared to analysis using 16S rRNA gene amplicons (16S), which are more mature, although they are limited by the resolution of the data. A typical 16S workflow is centered around amplicon sequence variants (ASVs) (12), which serve as the entries in a feature table. ASVs can be used to assign taxonomy for interpretation, but this taxonomic information is generally not used when calculating diversities, differential abundance, or in the statistics applied to those calculations. There is no assumption of the uniformity of the evolutionary level represented by ASVs, as they are independent of taxonomy (which is also not uniform in terms of the evolutionary period or diversity spanned by a taxon at a given rank). This property allows ASV feature tables to be combined with *de novo* and *a priori* phylogenetic inference methods (13), the latter of which benefit from precomputed reference phylogenies (14, 15), enabling statistical assessments that factor in evolutionary relationships informed by the molecular sequence data.

We suggest that the lessons learned from amplicon sequencing be applied to metagenomics. Thanks to the advances in efficient sequence alignment algorithms and the expansion of reference genome databases (16, 17) and phylogenomic trees (18, 19), it is now possible and increasingly preferable to develop a high-resolution, tree-structured data analysis strategy in shotgun metagenomics. In this work, we established and systematically evaluated a taxonomy-free analytical strategy, in which the features for analysis are individual reference genomes and the feature counts are the number of sequences aligned to these genomes. We refer to such features as operational genomic units (OGUs). This phrase, an echo of “operational taxonomic units” (OTUs) but replacing “taxonomic” with “genomic,” highlights the nature of the genome-based, taxonomy-free analysis. Meanwhile, “operational” indicates that this method does not rely on the direct observation of member genomes of the community (e.g., through metagenome assembly) but instead uses predefined reference genomes as a proxy to model the community composition. In this sense, OGUs are not analogs of ASVs. Instead, OGUs are the minimum units that can, from a technical standpoint, be produced by a reference-based method. An OGU table can be used to quantify the community structure and its relationship to biological traits. This process can be made more powerful with a reference phylogeny of the genomes, using tree-based methods such as UniFrac and phylofactorization (20).

The OGU method simplifies metagenomic data analysis by removing the bias of taxonomy, yet one can still describe the final results using taxonomic terms (as is done for ASVs). With minimum constraints, the OGU method achieves the finest-grained resolution in both the feature table and the relationship graph of features, as allowed by the reference. It produces biologically relevant results, which are robust against common artifacts in metagenomics and remain stable at very low sequencing depths. It should be noted that the OGU method is not meant to characterize the actual strain-level genetic variation in the community (as described in reference 21), nor does it attempt to infer the phylogenetic position of microbes (as described in reference 22). Instead, it operates within a reference-defined genome catalog and phylogeny, which subsequently facilitate comparing and combining results across studies.

Here, we describe an implementation of the method for generating OGU tables in the open-source bioinformatics tool Woltka. This program serves as a versatile interface connecting upstream sequence aligners (such as Bowtie2 and BLAST) and downstream microbiome analysis pipelines (such as QIIME 2). The program works seamlessly with the Web of Life (WoL) reference phylogeny that we previously developed to describe accurate evolutionary relationships among genomes (18), but it is not tied to a particular database or sequence aligner. In addition to a standalone command line interface, the package includes a QIIME 2 (11) plug-in to facilitate adoption and integration into existing pipelines. We have also made this method available through the Qiita web analysis platform (23), where it serves as the standard operating procedure for shotgun metagenomic data analysis, thereby enabling repository-scale processing and meta-analysis of metagenome data sets with OGUs. Thus far, we have applied the OGU method to reanalyze all public and private metagenomic data sets hosted on Qiita, totaling 64 studies, 369 preparations and 60,784 samples, as of January 2022.

Prototypes of the OGU method have already been used in multiple microbiome studies and have obtained biologically relevant results (e.g., see references 24–27). In this article, we introduce the principles and practices of the OGU method, demonstrate its efficacy in two real-world microbiome data sets, compare it with state-of-the-art metagenome analysis approaches and the alternative data type (i.e., 16S rRNA gene amplicons), and systematically evaluate its performance in various conditions. Given our findings, we propose the adoption of OGUs in the community ecology analysis of metagenomic data.

## RESULTS

### OGUs maximize resolution of community structures.

**FIG 1:**
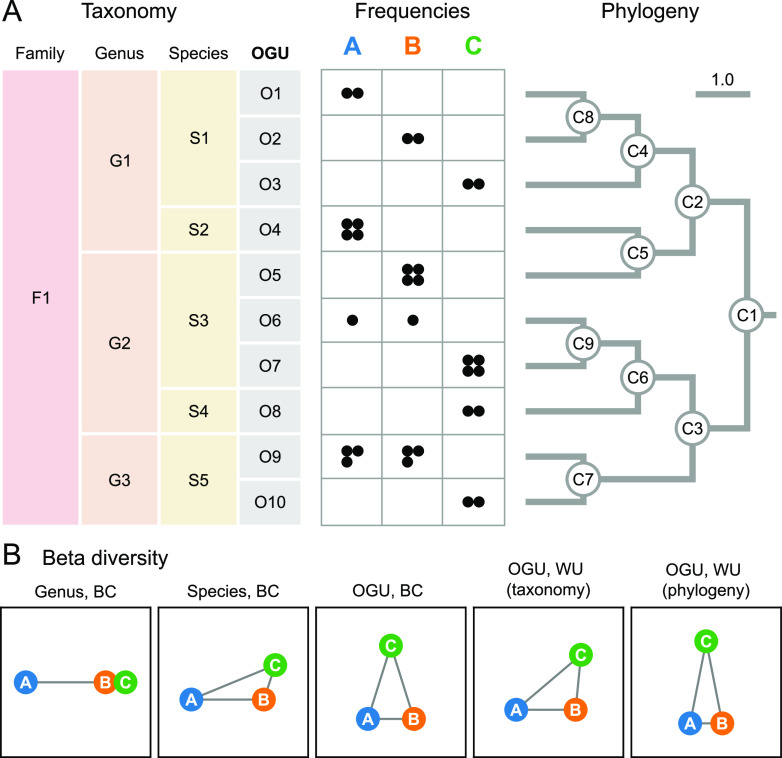
Feature resolution impacts community ecology analysis in small conceptual examples. (A) Synthetic data set involving three microbial communities, each having 12 unique read hits, as represented by black circles in the frequency table, to a total of 10 reference genomes (OGUs), classified under five species, three genera, and one family, as noted on the left. A phylogenetic tree of the 10 genomes is shown on the right. In this simplified case, the phylogeny is not much more complex than the taxonomy (with three more edges); however, the taxonomic assignment and the phylogenetic placement of genome O5 are not consistent. (B) Beta diversity of the data set. The three samples (circles) are connected by edges representing the pairwise distances calculated by Bray-Curtis (BC) or weighted UniFrac (WU) on the frequency table. For the latter measure, either the taxonomy or the phylogeny was used to quantify the hierarchical relationships among OGUs, as noted in the parentheses. The edge lengths were normalized so that their sum is equal in each graph. This synthetic case study demonstrates that different resolutions of features and feature structures can lead to very different conclusions regarding sample relationships.

The initial rationale and benefits of the OGU method are demonstrated with a synthetic example illustrated in Fig. 1. In this example, three metagenomes with 12 sequences each were aligned to 10 reference genomes, which were hierarchically organized by taxonomy (Fig. 1A, left) or by phylogeny (right). Beta diversity was calculated on feature tables at different levels, either on taxonomic units at the rank of genus or species or directly on reference genomes (i.e., OGUs), without the need for giving them taxonomic labels.

As demonstrated (Fig. 1B), the genus-level analysis, which had the lowest resolution (three genera), yielded spurious proximity between samples B and C, relative to sample A, largely determined by the differential abundance of genera G1 and G2. The species-level analysis with moderately higher resolution (five species) was able to bring A closer to B and C, mainly contributed by the identical frequencies of species S1, which could not be revealed at the genus level. The OGU-level analysis, having the highest resolution (10 features), revealed the separation between B and C due to distinct OGU composition, despite similar species counts (e.g., O5 and O7 have the same count within S3) and the proximity between A and B due to shared OGUs (O6 and O9). Additional structure was revealed by using the UniFrac metric, which considers the hierarchical relationships among features, hence further joining samples (here, A and B) sharing longer branches in the phylogenetic tree (even by different OGUs, such as O1 and O2) and separating those sharing shorter ones. Taxonomy may serve as a replacement for phylogeny, but it has a lower resolution than phylogeny (e.g., O1 and O2 are evolutionarily closer to each other than to O3, but taxonomy cannot reveal this) and sometimes does not reflect the true evolutionary relationships among organisms (e.g., O4 and O5 are here placed in different genera), which can impact the accurate modeling of community structures.

In summary, this example illustrates the potential benefit of increasing resolution in assessing the diversity of microbial communities. This “resolution” has two dimensions of meaning: first, the quantity of features representing individual microbiomes, and second, the granularity and accuracy of the structure that defines the relationships among individual features.

### OGUs enhance body environment and host sex-associated microbiome patterns.

**FIG 2:**
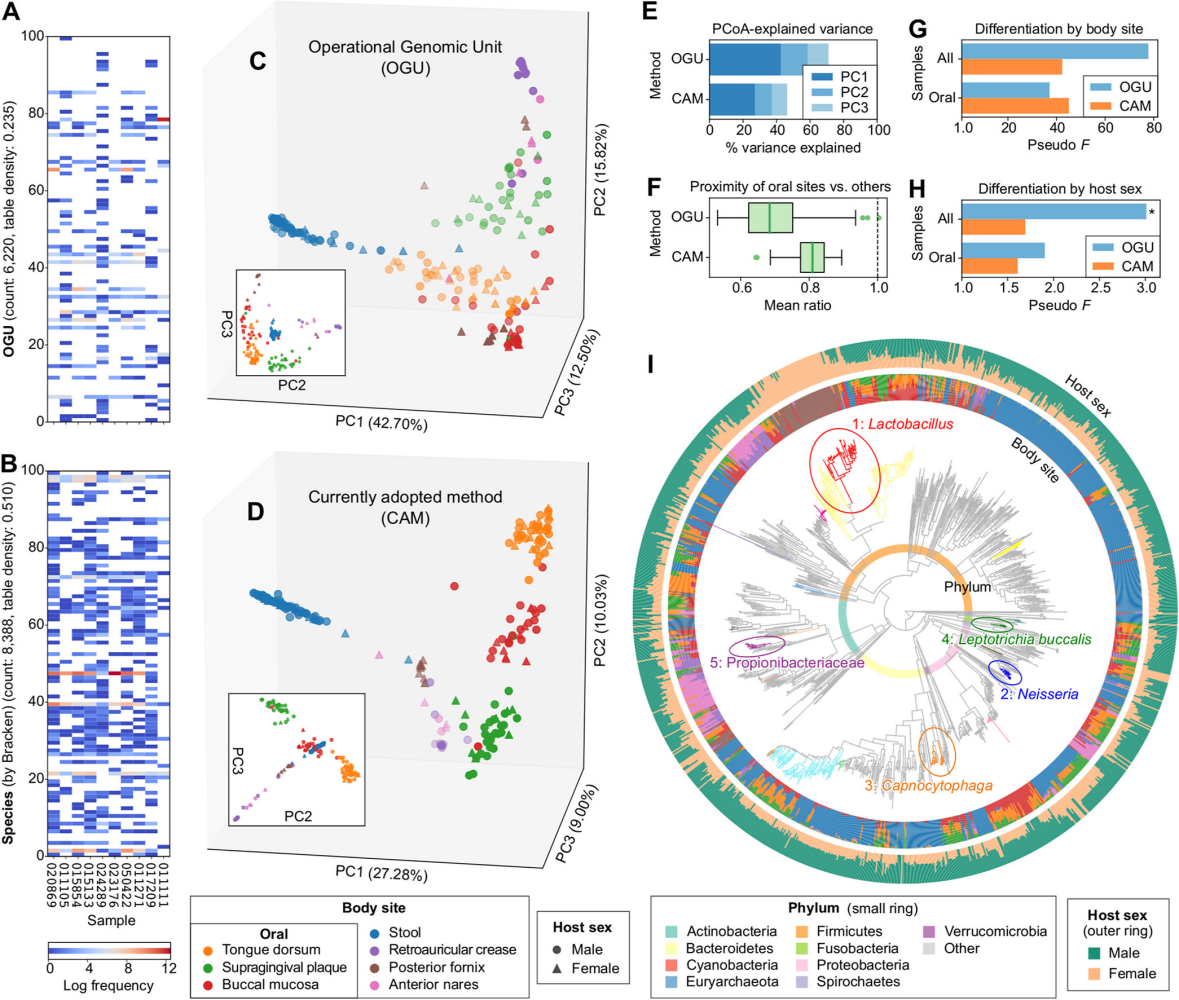
Analysis of the HMP metagenomes reveals clustering by body environment and differentiation by host sex. The analysis was performed on 210 samples subsampled to one million paired-end shotgun reads each. (A) Heat map of the OGU table randomly subsampled to 10 samples and 100 OGUs. (B) Heat map of a species-level taxonomic profile inferred by Bracken, subsampled to the same 10 samples and 100 randomly selected species. Empty cells represent zero values. The feature count and table density indicated in the *y* axis describe the entire (not subsampled) table. (C) PCoA based on OGUs using weighted UniFrac calculated with the WoL reference phylogeny based on the OGU table. Samples (dots) are colored by body site and shaped by host sex. (D) PCoA using the currently adopted method (CAM): Bray-Curtis calculated on species-level taxonomic units identified by Bracken, which shows a diagonal pattern that aligns all samples of the four nonoral body sites in one plane. (E) Proportions of community structure variance explained by the first three axes of PCoA. (F) Mean ratio of the beta diversity distances from any oral sample to a sample of the two other oral sites versus to that of nonoral body sites. The lower the mean ratio is, the more similar communities of the three oral sites are to each other in the background of multiple body environments. The bold line in each box represents the median. The whiskers represent 1.5 times the interquartile range (IQR). (G and H) PERMANOVA pseudo-*F* statistics indicating the differentiation of community structures by body site (G) and by host sex (H). The larger *F* is, the more distinct the community structures are between groups versus within groups. The *y* axis is aligned to an *F* value of 1.0 which indicates no difference. For panel G, all statistics have a *P* value of 0.001. For H, an asterisk indicates a *P* value of ≤0.05. (I) Differentially abundant phylogenetic clades by host sex inferred using PhyloFactor and visualized using EMPress on the WoL reference phylogeny. The tree was subsetted to include only OGUs detected in the data set. The top 20 clades by effect size are colored (full details provided in Fig. S1 and S2). The top five clades are numbered 1 through 5 by decreasing effect size, circled, and labeled with corresponding taxonomic annotations. The small color ring represents phylum-level annotations. The inner and outer bar plot rings indicate the OGU counts split by body site (using the same color scheme as in panels A and B) and by host sex, respectively.

Next, we demonstrated the typical use of the OGU method on the classic Human Microbiome Project (HMP) shotgun metagenomic data set (28), which contains 210 metagenomes sampled from seven body sites of male and female human subjects. We subsampled each metagenome to one million paired-end reads—a sampling depth close to the recommended lower threshold (500,000 reads) for “shallow” shotgun sequencing (29). The sequences were aligned to the WoL reference genome database (totaling 10,575 bacterial and archaeal genomes), and the alignments were processed using Woltka, resulting in an OGU table with 6,220 features (reference genomes) and a density of 0.235 (Fig. 2A). For comparison, a species-level taxonomic profile inferred by Bracken (30) using the same database has a feature count of 8,388 and a density of 0.510) (Fig. 2B).

10.1128/msystems.00167-22.1 FIG S1 PhyloFactor-inferred top 20 factors that differentiated samples by host sex in the HMP dataset. The explained variance (percent), *F* statistic, and *P* value of each factor are plotted. Tick labels are taxonomic annotations of the corresponding phylogenetic clades. Download FIG S1, PDF file, 0.03 MB. Copyright © 2022 Zhu et al. 2022 Zhu et al. https://creativecommons.org/licenses/by/4.0/ This content is distributed under the terms of the Creative Commons Attribution 4.0 International license.

10.1128/msystems.00167-22.2 FIG S2 PhyloFactor-calculated ILR sizes of the top 20 factors that differentiated samples by host sex in the HMP dataset. Numeric indices indicate the order of factors by effect size from high to low. Titles are taxonomic annotations of the corresponding phylogenetic clades. Two-tailed *t*-test *P* values of male versus female ILR sizes are also shown. Download FIG S2, PDF file, 0.6 MB. Copyright © 2022 Zhu et al. 2022 Zhu et al. https://creativecommons.org/licenses/by/4.0/ This content is distributed under the terms of the Creative Commons Attribution 4.0 International license.

Beta diversity analysis using the weighted UniFrac metric with the WoL reference phylogeny was performed on the OGU table. Principal coordinates analysis (PCoA) (Fig. 2C), with the first three axes explaining 71.01% of community structure variance (Fig. 2E), revealed that microbiomes were clustered mainly by the body site from which they were sampled, which overshadowed clustering by host sex, if any. This pattern is largely consistent with the previous report (28). For comparison, we analyzed the data set using Bray-Curtis on the species profile, a currently adopted method (CAM) (e.g., see reference 28). The PCoA plot by CAM (Fig. 2D), although with less explained variance (46.30%) (Fig. 2E), also displayed a clustering-by-site pattern. However, it is notable from the plot that sample clusters are aligned diagonally, a typical pattern indicating the saturation of distances caused by the inadequacy of shared features (species) among body sites (31) (Fig. 2D). This characteristic limits the power of resolving community diversity.

Permutational multivariate analysis of variance (PERMANOVA) of the beta diversity distance matrices suggested that both methods were able to clearly differentiate samples by body site (*P = *0.001), with the OGU method generating the stronger statistic (Fig. 2G) (OGU method, *F *=* *77.82; CAM, *F *=* *42.36). The distinction by host sex was less obvious. Only the OGU method was able to distinguish microbial communities based on sex (*F *=* *3.011, *P = *0.013), whereas the CAM failed to distinguish sex with statistical significance (*F *=* *1.692, *P = *0.086) (Fig. 2H). This demonstrated the power of the OGU method in capturing subtle but relevant trends, even when another primary factor (body site) can explain most of the variation in community diversity.

Three of the seven body sites are located in the oral environment: tongue, teeth and buccal mucosa (Fig. 2C and D). Together, they indicate weaker differentiation by sex (OGU, *F *=* *1.905, *P = *0.099; CAM, *F *=* *1.610, *P = *0.130) (Fig. 2H). We reason that sites sharing the same environment likely have higher microbial connections. To test this effect, we calculated the relative distance between the three oral sites versus that between oral sites and nonoral sites. This distance is significantly smaller with the OGU method (0.699 ± 0.098 [mean and standard deviation {SD}]) than with CAM (0.808 ± 0.051) (two-tailed paired *t* = −14.398, *P = *2.57e−26) (Fig. 2F), suggesting that OGUs are more effective at relating subgroups of samples with shared properties.

The OGU table plus the WoL tree further enabled differential abundance analysis using the phylogenetic factorization method (32) (Fig. S1 and S2). The result was visualized and analyzed using the recently released tree visualizer EMPress (33) (Fig. 2I). It revealed that the phylogenetic clade separated by factor 1 represents the genus *Lactobacillus*, contained in predominantly posterior fornix samples from female hosts, which was expected (34). Meanwhile, factor 2 (genus *Neisseria*), factor 3 (genus *Capnocytophaga*), and factor 4 (species Leptotrichia buccalis) are more frequently observed in the oral sites of male hosts. For comparison, we applied the tree-free method ANCOM (35) to the taxonomic profiles generated by alternative methods (Table S1). At the genus level, all four methods were able to capture only *Lactobacillus*, consistent with our factor 1. However, at species and OGU levels, results were discordant between methods, and no method reported any *Lactobacillus* sp. This exposed the limitation of confining analyses to one specific taxonomic rank without using phylogenetic information.

10.1128/msystems.00167-22.9 TABLE S1 Significantly differentially abundant taxa between samples from male and female hosts in the HMP cohort identified by the ANCOM method. Download Table S1, XLSX file, 0.01 MB. Copyright © 2022 Zhu et al. 2022 Zhu et al. https://creativecommons.org/licenses/by/4.0/ This content is distributed under the terms of the Creative Commons Attribution 4.0 International license.

### OGUs improve prediction of host age from the gut microbiome.

We next analyzed 6,430 stool samples collected through a random sampling of the Finnish population using both 16S rRNA gene amplicon sequencing and shallow shotgun metagenomic sequencing. This FINRISK study (36) provides an opportunity to explore the dependency of feature sets (e.g., taxonomic levels) and data source 16S rRNA amplicon versus shotgun metagenomic data) on the prediction accuracy of a machine learning model on a targeted phenotype (e.g., age). We measured the empirical error (mean absolute error [MAE]) in predicting human age using a random forest regressor (37), constructed using 5-fold cross-validation.

**FIG 3:**
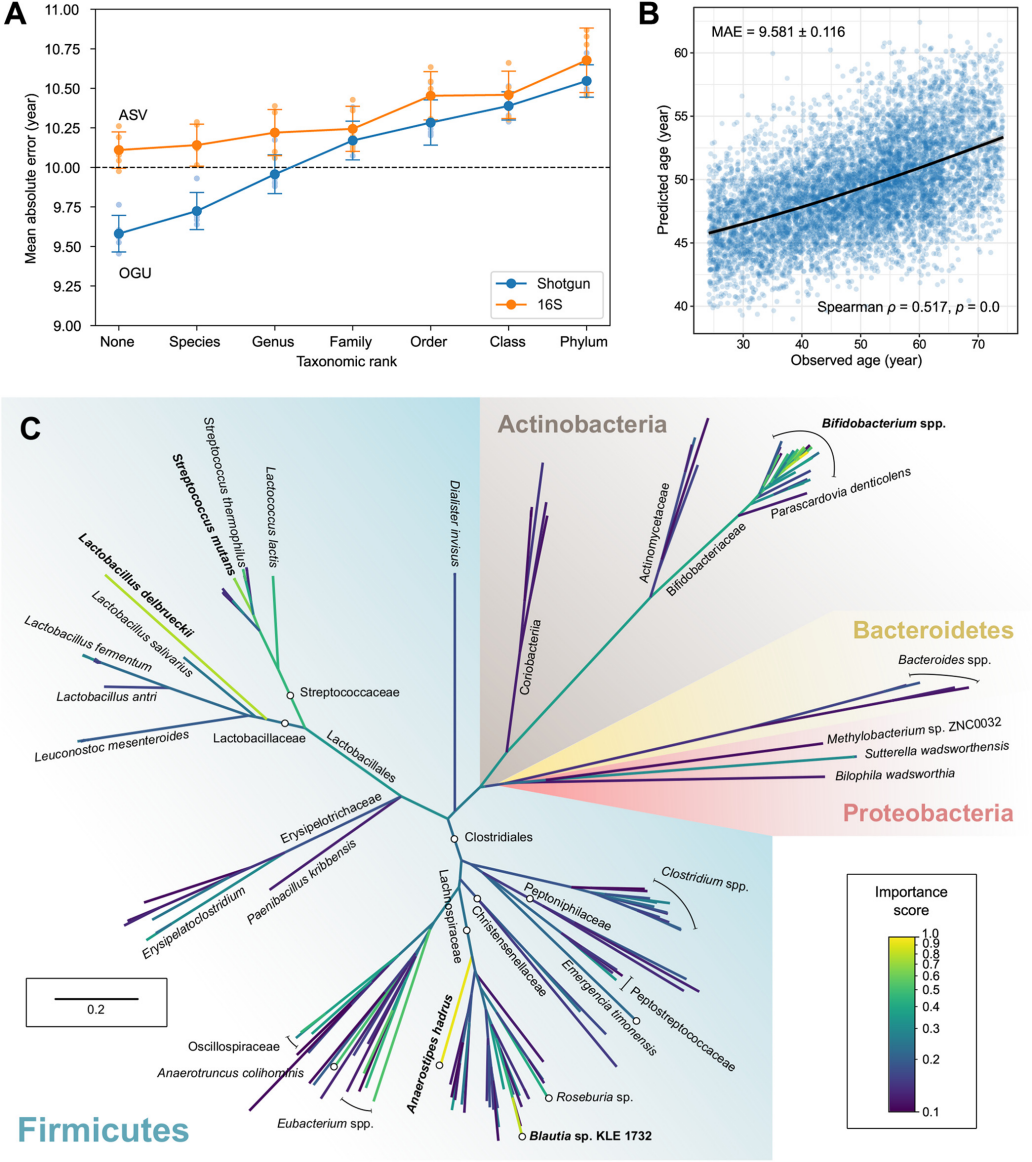
Analysis of the FINRISK metagenomes showing superior prediction accuracy over taxonomic units and 16S rRNA data. (A) The empirical error (MAE; a lower error indicates better performance) in predicting host chronological age using microbiome features at distinct taxonomic ranks in paired 16S rRNA amplicon and shallow shotgun metagenomics data with a random forest regressor. “None” represents the taxonomy-free, finest-possible level (ASV for 16S, OGU for shotgun). Small circles indicate MAEs in all iterations of 5-fold cross validation. Large circles and error bars indicate means and standard deviations of the five MAEs. (B) Scatterplot of the actual age versus the predicted age by the best-performing model with OGU features in the 5-fold cross-validation. The black line was generated using ggplot2’s local polynomial regression fitting. (C) Phylogenomic tree of 169 OGUs with importance scores of ≥0.1 in the prediction model. The tree was subsampled based on the WoL reference phylogeny and drawn to scale (branch lengths represent mutations per site). Branch colors indicate the mean importance score of all descendants of the clade. Taxonomic labels are displayed where needed. Circles and lines with stops are displayed where needed to assist location of taxonomic labels to target branches or clades.

Our results (Fig. 3A) showed the prediction accuracy continued to improve with lower microbial feature classification levels, resulting in lower absolute errors. Shotgun data outperformed 16S data at all levels and were able to reduce MAE to less than 10 years when analyzing at the genus level or below. Using OGUs, we were able to predict human age based on gut microbiome composition to an MAE of 9.581 ± 0.116 years (mean and SD) (Fig. 3B). ASVs, the finest possible resolution allowed by 16S data, resulted in a higher MAE of 10.110 ± 0.103 years (two-tailed *t* = −7.25, *P = *8.81e−5). Meanwhile, using the species-level profile inferred by Bracken on the shotgun data, we also obtained a higher MAE of 10.273 ± 0.089 years (versus OGU, two-tailed *t* = −10.59, *P = *5.53e−6) (Fig. S3). Decreasing sequencing depth did not reduce the age prediction accuracy for individual samples (Fig. S4). For example, samples with 320,000 to 366,000 metagenomic sequences (second bin from the low end in the figure) had an MAE of 9.290 ± 6.378 years, whereas samples with 1,386,000 to 1,931,000 sequences (second bin from high end) had an MAE of 10.118 ± 6.086 years, which were not significantly different (two-tailed *t* = −1.37, *P = *0.170). We then explored which OGUs contributed to the superior performance in age prediction compared to 16S rRNA ASVs. We identified a reduced set (*n *=* *128) of the most important OGUs that can maximize the prediction accuracy via a recursive feature elimination approach (Fig. S5).

10.1128/msystems.00167-22.3 FIG S3 MAE in predicting host chronological age from the FINRISK data set, using features at distinct taxonomic ranks inferred from shotgun metagenomic data using two tools: SHOGUN and Bracken. Both tools were able to reach species-level resolution, whereas the OGUs were inferred from the sequence alignments generated by SHOGUN, adding an extra, finer resolution to the tool. Small circles indicate MAEs in all iterations of fivefold cross validation. Large circles and error bars indicate the mean and standard deviations of the five MAEs. Download FIG S3, PDF file, 0.03 MB. Copyright © 2022 Zhu et al. 2022 Zhu et al. https://creativecommons.org/licenses/by/4.0/ This content is distributed under the terms of the Creative Commons Attribution 4.0 International license.

10.1128/msystems.00167-22.4 FIG S4 Prediction error by total frequency of OGUs per sample (which is equal to the number of sequencing reads that were aligned to any reference genome) in the age prediction model of the FINRISK dataset. Samples were binned such that each box consists of the same number of samples (429 per box) as sorted by sampling depth. Download FIG S4, PDF file, 0.02 MB. Copyright © 2022 Zhu et al. 2022 Zhu et al. https://creativecommons.org/licenses/by/4.0/ This content is distributed under the terms of the Creative Commons Attribution 4.0 International license.

10.1128/msystems.00167-22.5 FIG S5 MAE of age prediction models when using different numbers of the most important OGU features identified from the FINRISK data set. Download FIG S5, PDF file, 0.00 MB. Copyright © 2022 Zhu et al. 2022 Zhu et al. https://creativecommons.org/licenses/by/4.0/ This content is distributed under the terms of the Creative Commons Attribution 4.0 International license.

Among these important features, a few gut microbial lineages increased in relative abundance with aging, such as multiple OGUs assigned to Streptococcus mutans and *Eubacterium* sp. (Fig. 3C; Fig. S6 and S7). Remarkably, those Streptococcus spp. are typically located in the oral cavity but can be overrepresented in the gut of elderly individuals, suggesting potential microbial transmissions between oral and gut microbiomes related to typical aging in a large population (38, 39). Next, we also identified a few OGUs that were underrepresented in the elderly, such as ones assigned to Anaerostipes hadrus, Bifidobacterium longum, and Bifidobacterium saguini. In comparison, many important features identified in the 16S data had coarse taxonomic assignments, putatively because the partial sequences of a 16S rRNA gene cannot provide sufficient resolution. For example, a few ASVs annotated as the family *Lachnospiraceae* have been associated with aging in either this or past studies (40), whereas our method identified several OGUs under *Lachnospiraceae* that exhibited strong predictive powers for discriminating aging. The second most important OGU in the model was the genome of *A. hadrus* DSM 3319. It contains a 16S rRNA sequence that exactly matches the third most important ASV that was taxonomically annotated as *Lachnospiraceae* from the 16S data set. Nevertheless, it is important to understand that OGUs are close matches of sequencing data found in the reference database. One should not consider that the exact strains, such as *A. hadrus* DSM 3319, are present in the sample. Further investigation, such as metagenome assembly and genetic variation analysis, is needed when the end goal is to characterize microbial strains in the community.

10.1128/msystems.00167-22.6 FIG S6 Importance scores and taxonomic classifications of the top 32 OGU markers identified by the random forest regressor for age prediction on the FINRISK data set. Download FIG S6, PDF file, 0.04 MB. Copyright © 2022 Zhu et al. 2022 Zhu et al. https://creativecommons.org/licenses/by/4.0/ This content is distributed under the terms of the Creative Commons Attribution 4.0 International license.

10.1128/msystems.00167-22.7 FIG S7 Relative abundance of the top 32 OGU markers identified by the random forest regressor for age prediction in individual samples of the FINRISK dataset. Each panel is a scatter plot of predicted age versus reported age (same as Fig. 3B). Samples (points) are colored by the relative abundance of the OGU marker. Grey points indicate absence of the OGU marker. The blue lines were generated using ggplot2’s local polynomial regression fitting. Download FIG S7, PDF file, 1.3 MB. Copyright © 2022 Zhu et al. 2022 Zhu et al. https://creativecommons.org/licenses/by/4.0/ This content is distributed under the terms of the Creative Commons Attribution 4.0 International license.

### The OGU method is robust against common artifacts in metagenomics.

One major challenge that is unique to shotgun metagenomics is the ambiguity of sequence alignment and the prevalence of false-positive assignments that arise from it (41). Bioinformatically, it is challenging to match short DNA sequences to the reference genome that is the closest to their origin, and this challenge is amplified by the growing size of databases, which inevitably contain many highly similar genomes. Modern sequence aligners rely on heuristics in order to efficiently deal with very large reference databases and input data sets. They tend to report initial local optima without searching the entire database, which can lead to the inclusion of low-frequency, false-positive microbial taxa in the resulting profiles.

**FIG 4:**
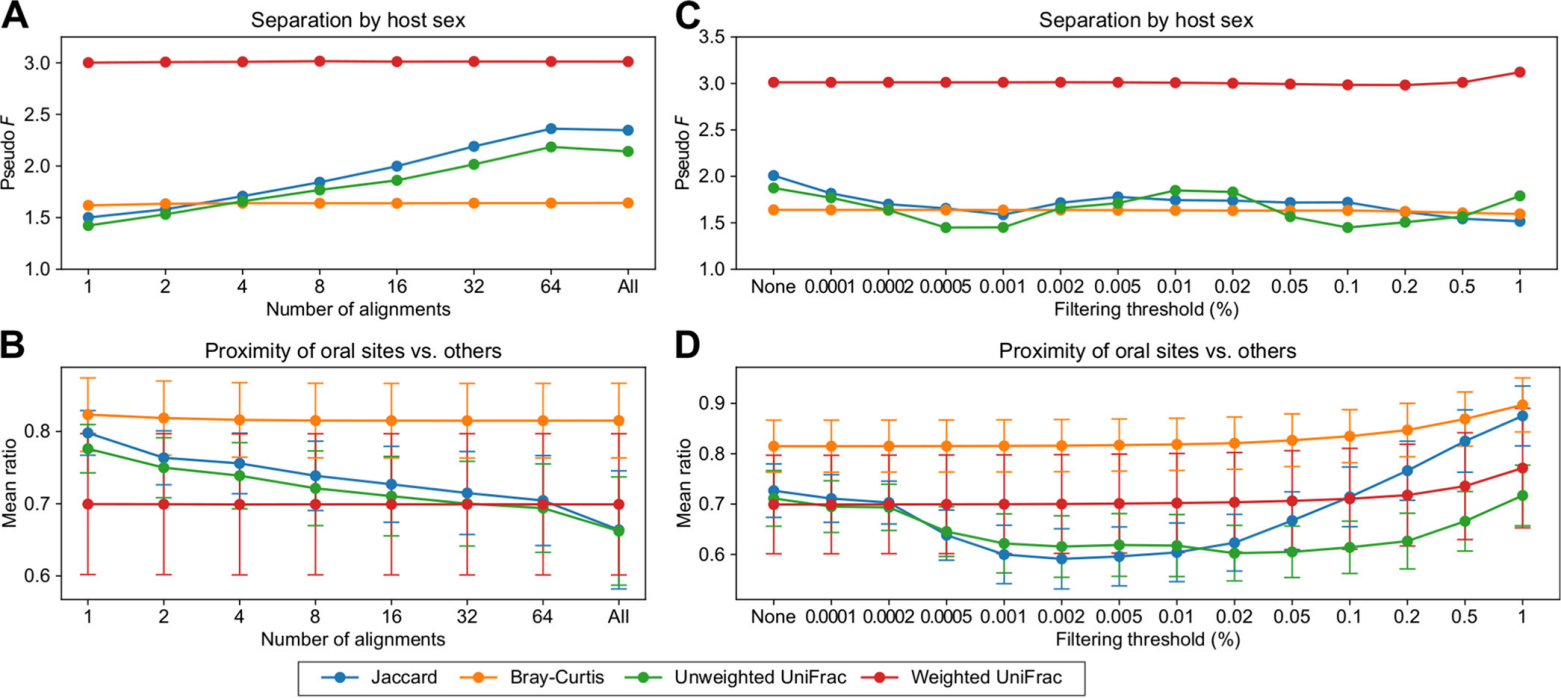
Impact of alignment artifacts on the result of the OGU analysis. (A to D) Robustness of analysis results on the HMP data set. Four commonly used beta diversity measures were tested: Jaccard (unweighted, tree free), Bray-Curtis (weighted, tree free), unweighted UniFrac (tree aware) and weighted UniFrac (tree aware). (A and B) Impact of the maximum number (*k*) of alignments to consider for each query sequence. This *k* value corresponds to Bowtie2’s “-k” parameter. “All” instructs Bowtie2 to return all possible alignments. (C and D) Impact of the feature filtering threshold. The original OGU table was filtered to remove OGUs that were below the per-sample relative abundance threshold indicated at the *x* axis. “None” indicates no filtering (original table). (A and C) PERMANOVA pseudo-*F* statistics for the separation between samples from male and female hosts. (B and D) Mean ratios of the distances from any oral sample to samples of the two other oral sites versus to that of nonoral body sites. The error bars indicate the means and standard deviations of the ratios.

In demonstrating the OGU method, we used SHOGUN to generate sequence alignments. This protocol reports up to 16 valid hits (*k* value) per query sequence. Next, instead of using complex methods such as the Bayesian framework (e.g., Bracken) to infer the most plausible taxa from ambiguous matches, the Woltka program retains and averages among all valid hits in constructing the OGU table (see Materials and Methods). We examined the effect of this parameter by varying the *k* value, i.e., 1, 2, 4, 8, 16, 32, and 64, and using a very slow exhaustive search mode. We found that the biological measurements inferred by OGUs remained almost unchanged for all values of *k* when an abundance-based metric (weighted UniFrac or Bray-Curtis) was used (Fig. 4A and B). Meanwhile, using presence/absence-based metrics (unweighted UniFrac and Jaccard) resulted in inconsistent results, suggesting cautionary use in the OGU analysis.

Previous work has demonstrated that bioinformatic efforts to remove erroneous assignments have limited efficacy (41). Given a profile that could contain many unidentified false positives, a typical approach is to consider only taxa with a relative abundance above a given threshold in each sample (41). We also provide this function in Woltka to facilitate the user’s preferences. However, this workaround indiscriminately loses information from low-abundance taxa that may represent biologically important signals. We further investigated whether filtering an OGU table has an impact on beta diversity by testing a gradient of filtering thresholds and found the measurements to be largely stable when using an abundance-based metric (Fig. 4C and D). They began to moderately change only at extremely high filtering thresholds (0.1 to 1%). Meanwhile, the measurements diverged notably when a presence/absence-based metric was used, as described above.

These analyses suggest that the OGU method is robust against potential alignment ambiguity and false-positive assignments, as long as the abundance of OGUs is taken into consideration, which effectively smooths out noise. This simplifies the analytical workflow and removes the dilemma between data preservation and data modification.

### OGUs are useful indicators of community composition.

An important goal of microbiome studies is to assess community composition. Here, we asked whether the taxonomic units indicated by individual OGUs actually exist in the community and to what extent an OGU table can be interpreted as community composition.

**FIG 5:**
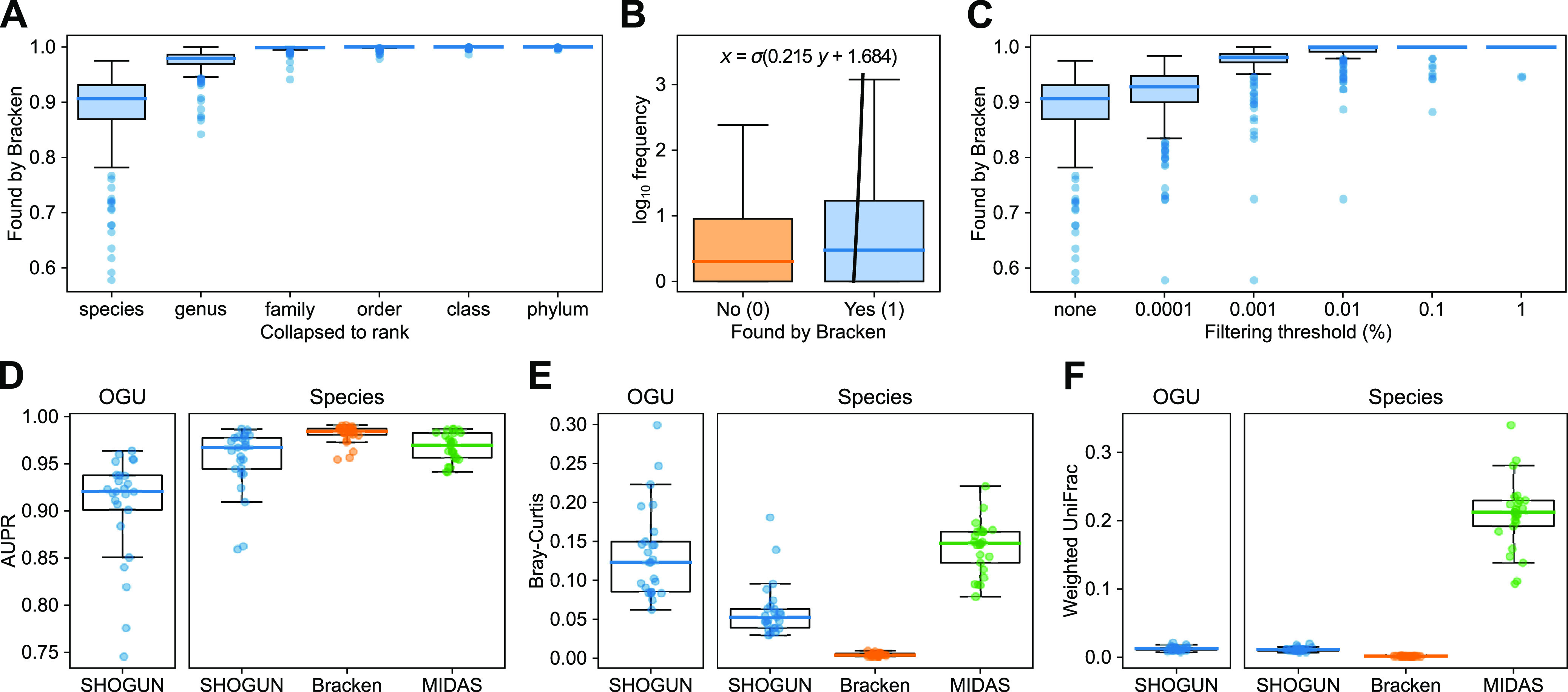
Relationship between OGU taxonomy and community composition. (A to C) Presence of OGU-informed taxonomic units in Bracken-inferred taxonomic profiles using the HMP data set (*n *=* *210). (A) Per-sample fraction of OGUs whose corresponding taxonomic units at different ranks were found by Bracken. (B) Presence/absence of corresponding species of all OGUs in all samples (*n *=* *307,060) in the Bracken result. The *y* axis indicates the frequency of individual OGUs per sample (out of 1 million paired-end reads). Outliers are not displayed due to the sample size. The bold line is a logistic regression curve, with coefficient and intercept annotated. (C) Per-sample fraction of OGUs filtered by different relative abundance thresholds whose corresponding species were found by Bracken. (D to F) Evaluation of the accuracy of OGU or species assignments and abundance estimation using a simulated metagenomic data set (*n *=* *25) by the area under the precision/recall curve (AUPR) (D), Bray-Curtis (E), and weighted UniFrac (F) against the ground truth. Three tools—SHOGUN, Bracken, and MIDAS—were evaluated. The bold line and the whiskers represent median and 1.5 times the IQR, respectively. Each dot represents a simulated metagenomic sample.

To address these questions, we first tested the congruence between OGU-informed taxonomic units and the taxonomic profile inferred by Bracken within the 210 HMP metagenomes. Note that the Bracken result served as a reference one would expect from a typical taxonomic profiler, instead of ground truth. The result showed that the corresponding species of 88.78% ± 7.04% (mean and SD) OGUs were also found by Bracken. This ratio increased at higher taxonomic ranks (Fig. 5A), with 97.38% ± 2.24% at the genus level and 99.76% ± 0.57% at the family level. On the other hand, the corresponding species of OGUs with higher abundance had a higher chance of being found by Bracken (Fig. 5B). After removal of low-abundance OGUs, we observed a further increase of the ratio (Fig. 5C). For example, when OGUs with a per-sample abundance less than 0.01% were removed, the corresponding species indicated by 99.07% ± 2.43% of the remaining OGUs were found by Bracken.

We note that the HMP data set does not allow direct evaluation of assignment accuracy as the ground-truth is not known. Therefore, we next generated a simulated microbiome data set for which we could verify ground-truth assignments, consisting of 25 samples representing four human body sites and the built environment (see Materials and Methods). The OGU assignments had an area under the precision/recall curve (AUPR) of 0.905 ± 0.057 (mean and SD), which was moderately lower than that of the species-level assignments (0.954 ± 0.034) (Fig. 5D). This was largely anticipated, since OGU represents a lower classification level than species. This difference was also reflected by the greater Bray-Curtis dissimilarity from the true community structure (OGU, 0.1346 ± 0.0591; species, 0.0615 ± 0.0345) (Fig. 5E). However, when phylogeny was accounted for, the weighted UniFrac distances were close to zero and to each other (0.0128 ± 0.0032 versus 0.0115 ± 0.0030) (Fig. 5F), suggesting that phylogeny-aware metrics are more robust for beta diversity analysis based on OGUs than phylogeny-agnostic ones.

These results suggest that we can relatively confidently discuss the taxonomic composition and individual components of the community based on the taxonomic assignments of OGUs. They also provide a useful reference for the user to decide the taxonomic rank and filtering threshold to adopt when they want to confidently make these discussions (for example, OGUs with 0.01% or higher relative abundance have 99% probability to match species found by Bracken, according to Fig. 5C). The last result (Fig. 5E) further demonstrates the efficacy of phylogeny-aware analysis in approximating true community composition, in spite of potential imprecise assignments.

### The OGU method is generally effective with flexible protocol design.

**FIG 6:**
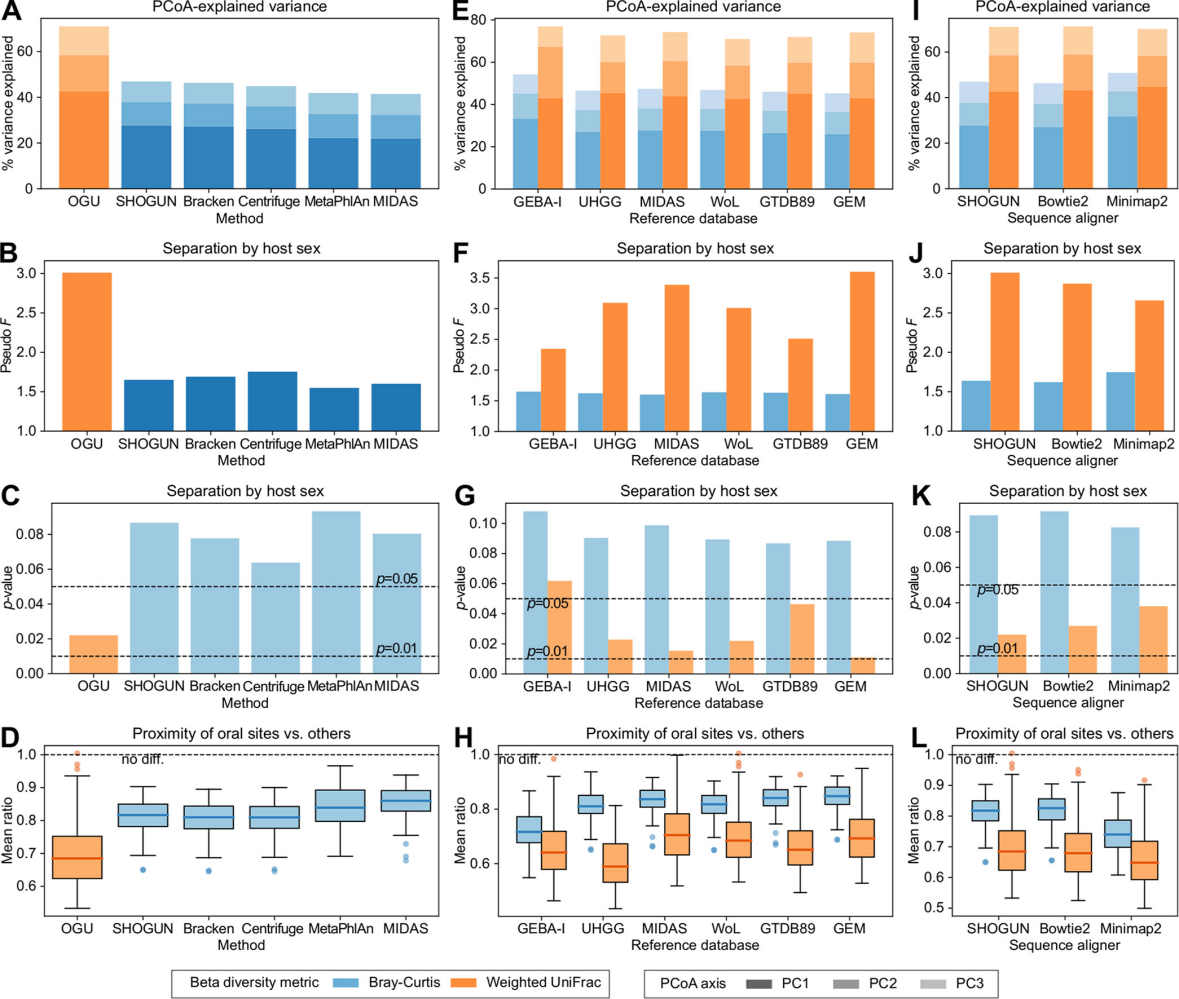
Comparison of beta diversity analysis results on the HMP data set using alternative protocols. (A to D) Comparison of the OGU method with five taxonomic profilers. The OGU table was extracted from SHOGUN’s alignment file using Woltka and analyzed using weighted UniFrac with the WoL reference phylogeny. It was compared with species-level profiles analyzed using Bray-Curtis. (E to H) Comparison of six reference genome databases used for generating the OGU table. The six databases are ordered by their volume from small (left) to large (right). Each OGU table was analyzed using either Bray-Curtis or weighted UniFrac with the original phylogenetic tree provided in the database. (I to L) Comparison of three sequence aligners used for generating the OGU table. (A, E, and I) Proportions of variance explained by the first three axes of PCoA. (B, F, and J) PERMANOVA pseudo-*F* statistics for the separation between samples from male and female hosts. (C, G, and K) *P* values of the corresponding PERMANOVA tests. (D, H, and L) Mean ratios of the distances from any oral sample to samples of the two other oral sites versus to that of nonoral body sites. The bold line and the whiskers represent median and 1.5 times the IQR, respectively.

Although we have demonstrated the advantage of a specific protocol for generating and analyzing OGUs over one example of currently adopted methods (Bracken), one may ask whether this outcome holds true when alternative methods are used. Here, we extended the comparative analysis of the HMP data set to include a total of five taxonomic profilers, representing two mainstream strategies for analyzing metagenomic data: SHOGUN (42), Bracken (30), and Centrifuge (4) utilize the information of whole genomes and report sequence abundances, whereas MetaPhlAn (43) and MIDAS (10) use certain sets of phylogenetically informative marker genes and report taxonomic abundances (for a critical review of the distinguishment of the two, see reference 44). The results showed that regardless of the taxonomic profiler of choice, the Bray-Curtis analysis at the species level consistently failed to match the resolving power of that using weighted UniFrac on the OGU table (Fig. 6A to D).

Because OGUs are reference genomes that serve as proxies for microbial diversity in the sample, one may postulate that the effectiveness of the OGU method is largely influenced by the content of the reference genome database. To test this effect, we performed the OGU analysis using five established catalogs of microbial genomes: GEBA-I (45), UHGG (9), MIDAS (10), GTDB89 (8), and GEM (46), in addition to the WoL database. These six databases represent a gradient of volumes (genome count and total size) and a variety of inclusion foci (see Materials and Methods). Importantly, they each include a reference phylogeny inferred by the original authors (despite using different approaches), which we used in the OGU analysis. The results showed that the phylogeny-aware method (weighted UniFrac) always outperformed the phylogeny-agnostic method (Bray-Curtis), regardless of the database (Fig. 6E to H). Except for the smallest database (GEBA-I), all databases were able to distinguish samples of the two sexes in the HMP cohort with statistical significance (*P* ≤ 0.05). The *F* statistic varied from 2.51 to 3.60, which is likely influenced by both the genome pool and the phylogenetic tree. In contrast, Bray-Curtis failed to capture this separation for any database, and interestingly, its *F* statistics were fixed within a very small range (1.60 to 1.65). This result suggests that the OGU method is compatible with many databases of diverse microbial genomes, while enabling superior phylogenetic comparisons of metagenomic data.

Given that OGUs are derived from the alignments of sequence data against reference genomes, the choice of the alignment algorithm may also play a role. Our default protocol used a specific Bowtie2 parameter set implemented in SHOGUN that was optimized for shotgun metagenomics (see Materials and Methods). Hence, we tested Bowtie2 with its default parameters, and an alternative aligner, Minimap2 (47), also using its default settings. The results showed again that weighted UniFrac outperformed Bray-Curtis for all three criteria considered (Fig. 6I to L). Separation by host sex was consistently significant (*P < *0.05), although the *F* statistic varied moderately (2.66 to 3.01). This result suggests that the OGU method is flexible with respect to the alignment method while again enabling superior phylogenetic analysis.

### The OGU method remains effective at very low sequencing depths.

Shotgun metagenomics is advantageous over amplicon-based studies because of its higher resolution and the capability of informing genetic variations; however, the experimental cost is orders of magnitude higher. A new strategy, “shallow shotgun metagenomics,” was proposed in recent years, suggesting that as few as 500,000 sequencing reads per sample is sufficient for resolving microbial composition and studying community ecology (29). This strategy significantly reduces the expense of metagenomic sequencing, therefore enabling studies at an unprecedented scale.

**FIG 7:**
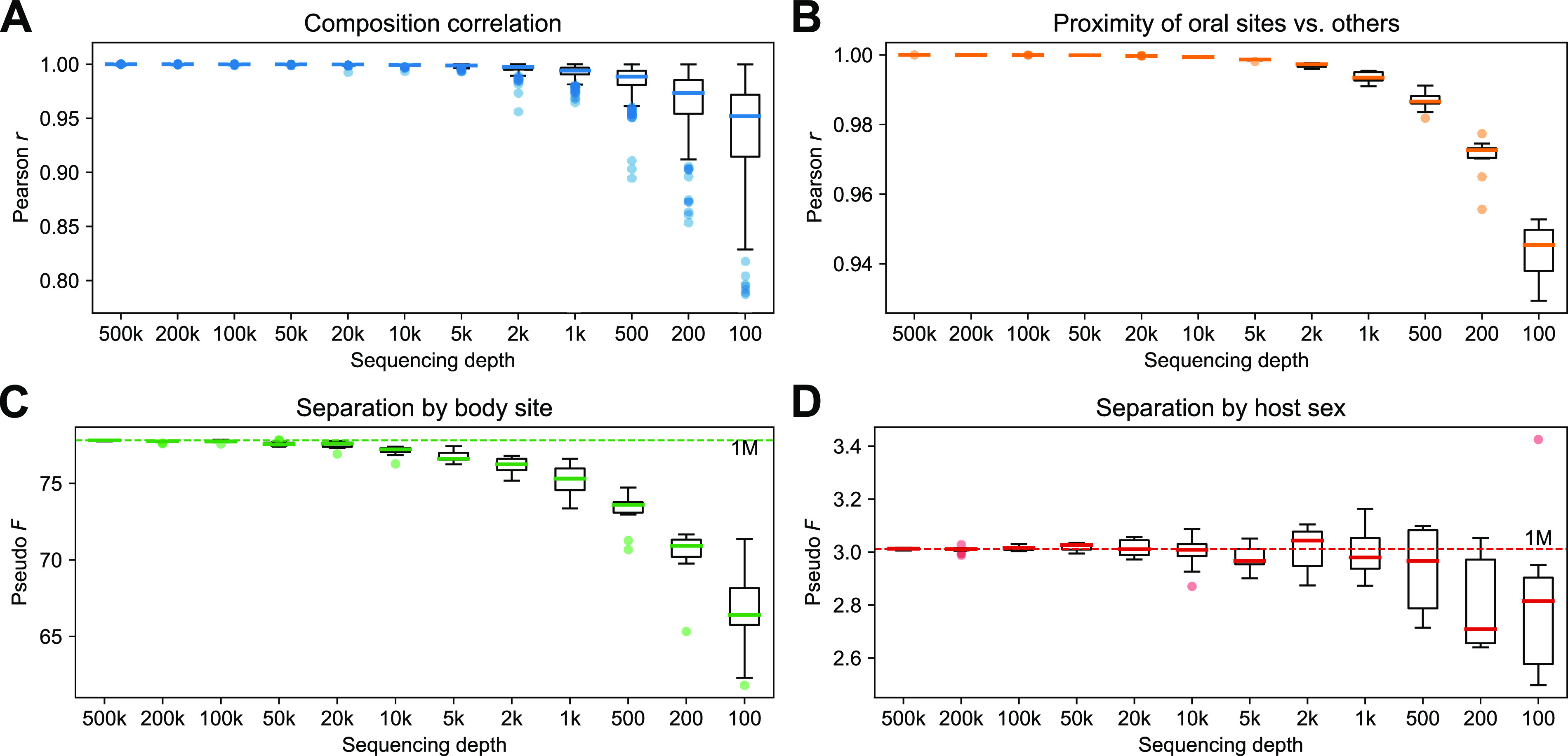
Impact of sequencing depth on the result of the OGU analysis. The original OGU table generated from 1 million paired-end reads of the HMP data set was randomly subsampled to each of the sampling depths indicated at the *x* axis, which is equivalent to the number of paired-end reads in the original sequencing data (see Materials and Methods). (A) Pearson correlation coefficient (*r*) between the composition (frequencies) of the original OGU table and the subsampled tables. To ensure visual resolution of the boxes, three outliers of 1,000, 200 and 100, respectively, are not visible in the range of the *y* axis. (B) Pearson’s *r* between the ratios of distances from any oral sample to sample of the two other oral sites versus to that of nonoral body sites. (C and D) PERMANOVA pseudo-*F* statistics on body site (C) and host sex (D). The results presented in panels B, C, and D were calculated from 10 replicates of random subsampling at each depth. The dashed lines indicate the statistic calculated on the original table. In all panels, the bold line in each box represents the median. The whiskers represent 1.5 times the IQR. Note that the ranges of the *y* axes differ between panels.

To assess the efficacy of the OGU method in the framework of shallow shotgun metagenomics, we reran the HMP analyses along a gradient of decreasing sequencing depths (Fig. 7). The correlation between the original OGU table (from one million read pairs) and each of the subsampled OGU tables was consistently high. A Pearson’s *r* of 0.961 ± 0.0726 (mean and SD, same below) was retained even at the sequencing depth of 200 (unit: read pairs, same below) (Fig. 7A). The oral-versus-other relative distance (see above) retained a Pearson’s *r* of 0.971 ± 0.00613 when sampling depth was 200 (Fig. 7B). The PERMANOVA pseudo-*F* statistics were close to the original statistic and remained largely stable down to very low sequencing depths. The mean difference from the original statistic was still within 5% at the sequencing depth of 1,000 for body site (3.349 ± 1.361, percentage of the original statistic), or 500 for host sex (2.680 ± 5.473) (Fig. 7C and D). These findings suggest that the OGU method remains valid even with very shallow metagenomic samples, including those that would otherwise be considered unusable for typical metagenomic analyses.

For comparison, we performed the same test using two taxonomic profilers, Bracken and MIDAS, representing the two main strategies widely adopted for this task (see above). The results (Fig. S8) suggested that the whole-genome-based profiler Bracken was also able to produce stable estimates of biologically relevant metrics, although moderately less so than the OGU method (for example, the *F* statistic for body site decreased by nearly 20% [17.990 ± 0.4062] at a sequencing depth of 1,000). In contrast, results of the marker gene-based profiler MIDAS showed the *F* statistic to decrease rapidly at lower sequencing depths, an expected pattern because marker genes constitute only a very small proportion of the microbial genomes and are therefore more sensitive to reduced sequencing depth.

10.1128/msystems.00167-22.8 FIG S8 Impact of sequencing depth on the analysis results of the HMP dataset. Three methods—OGU (identified by SHOGUN), Bracken, and MIDAS—were tested. Intermediate files of the three analyses were randomly subsampled to simulate the sequencing depths indicated at the *x* axis (unit: number of paired-end reads) (see Materials and Methods). (A) Pearson correlation coefficient (*r*) between the composition (frequencies) of the original result and the subsampled ones. (B) Pearson’s *r* between the ratios of distances from any oral sample to sample of the two other oral sites versus to that of nonoral body sites. (C and D) Ratio of PERMANOVA pseudo-*F* statistics on body site (C) and host sex (D) of subsamples versus the original result. The error bars indicate the means and standard deviations of the ratios. The dashed lines represent the same metric at the full depth (1 million). The results in panels B, C, and D were calculated from 10 replicates of random subsampling at each depth. The Bracken run frequently crashed at a depth of 200, so data of 200 and below were not shown. The MIDAS analysis failed to generate results at lower depths because one or more samples received all-zero species abundances after subsampling. Specifically, 9 out of 10 replicates succeeded at a depth of 20,000, 7 at 10,000, and 5 at 5,000. Data are not shown at depths below 5,000. Download FIG S8, PDF file, 0.1 MB. Copyright © 2022 Zhu et al. 2022 Zhu et al. https://creativecommons.org/licenses/by/4.0/ This content is distributed under the terms of the Creative Commons Attribution 4.0 International license.

### Underlying rationales for taxonomy-independent microbiome analysis.

Here, we explored the intrinsic reasons why OGUs with phylogenetically informed approaches confer significant advantages when microbial communities are analyzed. Broadly, this explanation can be broken down into two parts: feature extraction (the minimal classification units of microbial components in a profile) and feature structuring (the relationship graph among the classification units that guides subsequent analyses). With the OGU method, we advocate using the finest-possible resolution of features that is allowed by read-based metagenomics, i.e., individual reference genomes, and the finest-possible structure of the features, i.e., a phylogenetic tree, while bypassing taxonomy as a necessary component in conventional solutions.

It is important to understand what role taxonomy plays in this framework. Taxonomy, despite being coarse-grained and error-prone in describing evolutionary relationships (compared with phylogeny), provides an operational and hierarchical method for classification. Taxonomy can be utilized in an analysis in two ways. First, microbes are grouped into taxonomic units on a selected rank (e.g., species or genus)—an operation which itself bears, albeit implicit, consideration of evolutionary relationships—followed by analyses that treat all taxonomic units on this rank equally (i.e., without structure). Second, the taxonomic tree itself can serve as a limited replacement of phylogeny to drive UniFrac and other tree-aware analyses. In this context, branch lengths are not weighted by an evolutionary distance between nodes but instead fixed at a constant value between adjacent taxonomic ranks. This strategy, although less common, has been adopted in previous works (e.g., see reference 48).

**FIG 8:**
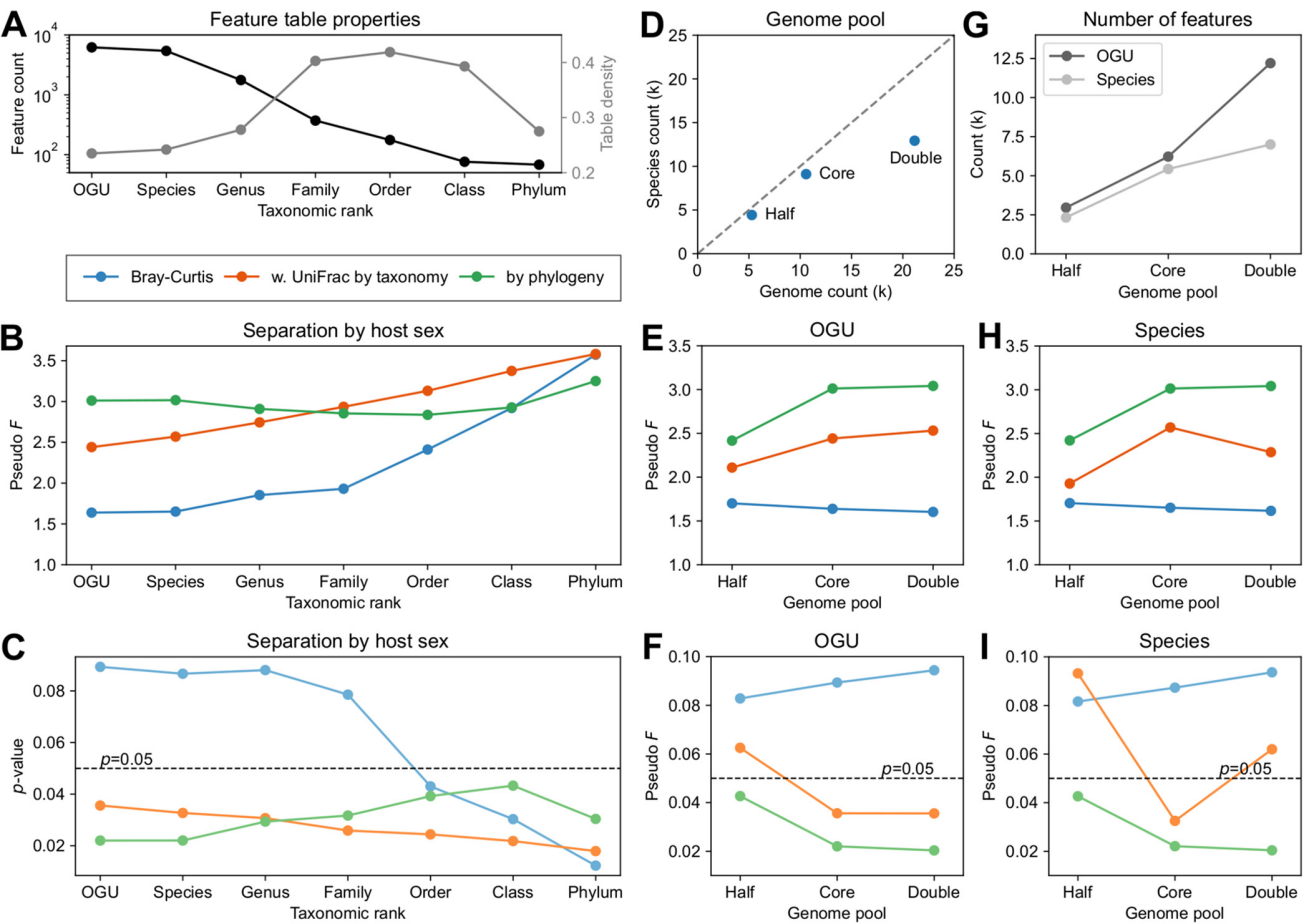
Impact of classification resolution and reference genome pool on the analysis results on the HMP data set. All feature tables were generated using the SHOGUN/Woltka protocol. (A to C) Comparison of community profiles classified to OGUs or each of the seven standard taxonomic ranks. (A) Feature counts and table densities of the profiles. (D to I) Comparison of OGU-level and species-level profiles generated using a gradient of sizes of the reference genome pool sampled from the same genome collection. (E) Numbers of genomes and species in the three genome pools: “half,” “core,” and “double.” Note that “core” is the original WoL database, and it is a superset of “half” and a subset of “double.” (I) Numbers of OGUs and species classified in the profiles. (B, E, and H) PERMANOVA pseudo-*F* statistics for the separation between samples from male and female hosts. (C, F, and I) *P* values of the corresponding PERMANOVA tests.

Using the HMP data set, we systematically tested and compared various options for feature extraction, i.e., OGUs, and each of the six standard taxonomic ranks from species to phylum, and for feature structuring, i.e., weighted UniFrac with phylogeny or with taxonomy, or Bray-Curtis, the last of which is equivalent to weighted UniFrac with a completely unresolved tree and an even sample size. The result (Fig. 8A to C) was interesting in several respects. First, the phylogeny-aware analysis yielded relative stable estimates of the degree of separation by host sex at most ranks (Fig. 8B). In comparison, results of the taxonomy-based analysis and the tree-free (Bray-Curtis) analysis continuously change by rank from low to high (Fig. 8B). Bray-Curtis produced the most unstable results across ranks: from OGU to family, it failed to separate samples with statistical significance (Fig. 8B and C). Taxonomy’s results were intermediate compared to the other two methods at lower ranks (OGU, species, and genus).

It is also noticeable that the phylogeny-based analysis at the OGU and the species levels yielded very similar results (Fig. 8B and C). One possible explanation is that the WoL database used in the default protocol has 10,575 genomes classified to 9,105 species (1.16 genomes per species on average). This provides a limited increase of resolution below the species level. One may further reason that, as the database size increases, the gap between species and OGUs will become more notable. To test this hypothesis, we created two additional databases, containing half or double the number of genomes of the original WoL database (“core”). As one would expect, the “double” database provided higher subspecies resolution (1.64 genomes per species on average) (Fig. 8D).

The result (Fig. 8D to I) showed that the phylogeny-based analysis produced similar results between the core and double databases, whereas that of the half database differed from the other two and was statistically less significant. This pattern held when either OGUs or species were analyzed. The immediate implication is that the current WoL database (core) is likely sufficient for this study, as adding more strain-level biodiversity did not have a strong impact, as reducing biodiversity did. On the other hand, the taxonomy-based analysis produced slightly more diverging results when OGUs were used and notably more diverging results when species were used. Finally, Bray-Curtis consistently failed to produce statistically significant results.

These two analyses point to a consistent conclusion: (i) diversity analyses using an explicit phylogenetic tree tend to generate stable estimates of biologically relevant measurements that are robust against the choice of the classification level of features, and (ii) relying on taxonomy has some merits, but it is heavily influenced by how taxonomic information is utilized.

## DISCUSSION

The OGU method introduced in this article provides a way to maximize the resolution of feature tables by directly considering reference genomes without relying on taxonomic classification in shotgun metagenomics studies. Although the strategy of taxonomy-independent analysis of community structure has been widely adopted in 16S data analysis, it remains underexplored in metagenomics. Our study shows that sequence alignment hits to individual reference genomes can be used as the minimum unit for features, referred to here as OGUs. Through comparative analysis of OGU and alternative methods using a synthetic case study and two real-world microbiome studies, we demonstrated that multivariate statistics and machine learning methods developed and matured in the field of 16S rRNA gene amplicon analysis can be directly applied to OGUs to provide biologically relevant insights. The OGU results often are superior to currently adopted metagenomic classification methods and ASV analysis of the 16S rRNA data. Meanwhile, we showed that the use of taxonomic units as features, as many researchers have been doing to date, has conceptual and performance limitations compared with the OGU method.

The independence from taxonomy further facilitates the utilization of phylogenetic trees. A researcher can choose from precomputed reference phylogenies, such as the one we introduced in the Web of Life (WoL) project (18), or custom phylogenomic trees computed from *de novo* construction or placement, through tools such as PhyloPhlAn3 (22) and DEPP (49), which are scalable to large numbers of genomes. This connects evolutionary biologists’ efforts in updating the tree of life (e.g., see references 18, 19, and 50), computational biologists’ efforts in forging phylogeny-aware methods (e.g., UniFrac and Phylofactor), and microbiome scientists’ pursuits of relating high-dimensional microbiome data with biology. On the other hand, although there have been remarkable efforts toward curating taxonomy using phylogenetics, the number of taxonomic ranks is limited (typically 7 to 8) and can constrain the topology for an ever-growing number of sequenced genomes. The history of 16S rRNA studies (13) is repeating itself in whole-genome studies, such that building a phylogeny is not only advantageous but often more feasible than defining taxonomy, and the OGU method powerfully provides an analogous extension to shotgun sequencing studies.

**FIG 9:**
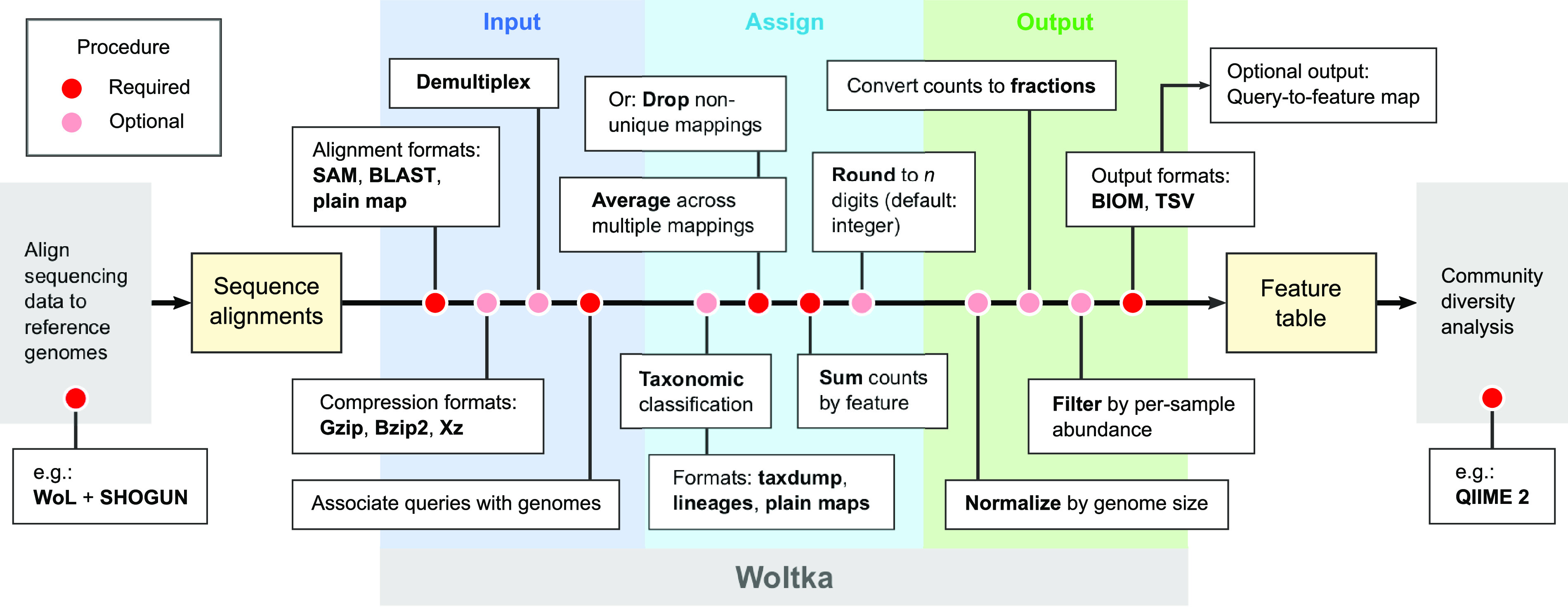
Procedure for generating OGU tables, as implemented in Woltka. This program serves as an interface connecting upstream sequence alignment and downstream community diversity analysis. The inputs are sequence alignments, and the outputs are feature tables (in this case, OGU tables). The pipeline contains multiple procedures, with rich format support and flexible options to address diverse user needs.

Our systematic investigation of the performance of OGUs in various conditions showed that they consistently produce biologically relevant results, as long as a phylogeny-aware and abundance-based metric (such as weighted UniFrac) is used. The results are robust to alignment ambiguity and low-abundance assignments, which are common challenges in shotgun metagenomics. The PERMANOVA statistics remain stable at very low sequencing depths, potentially permitting even shallower metagenomic sequencing in microbiome research while still deriving biologically conclusive results. The OGU method is not only effective with the recommended protocol (WoL + SHOGUN), but it is also flexible to the choice of sequence aligners and reference genome catalogs or phylogenies (Fig. 6), thus minimizing user efforts to integrate the OGU method into existing data analysis pipelines. Furthermore, the adoption of the OGU method can be conveniently achieved through the function-rich, highly customizable pipeline (Fig. 9) implemented in our software package Woltka.

Although showing its power for general metagenomics studies, our method so far has not provided definitive solutions to several practical questions, which remain to be further investigated. First, we showed that diversity metrics such as Jaccard and unweighted UniFrac, which rely on the presence/absence of features instead of their abundances, produced less stable results when alignment count or filtering threshold changed (Fig. 4). This issue is likely associated with the “noisy” nature of shotgun metagenomics, as discussed above. Future efforts may focus on the improvement of feature extraction through better handling of alignment ambiguity and also filtering to reduce off-target hits, both of which have been successful in other applications (41) but which we believe merit a detailed discussion that is outside the scope of the present work. Second, we recognize that this work does not address alpha diversity directly; however, an important consideration is that the currently most widely adopted alpha diversity metric that considers phylogeny, Faith’s PD (51, 52), is based on the presence/absence of features and will be impacted by alignment ambiguity (see above). A thorough investigation of the impact of feature abundance (e.g., see reference 53), alignment ambiguity, feature filtering, and other factors will be necessary to point to a robust method for estimating the alpha diversity of metagenomes in a phylogenetic context. Meanwhile, multiple nonphylogenetic, abundance-based metrics (such as Shannon index) already exist for deriving alpha diversity estimates from shotgun metagenomic data and have been recently benchmarked (44).

Third and most importantly, the robustness of an OGU analysis is limited by the comprehensiveness of the reference database. Although available genomic data have grown to an enormous volume, the size of a reference genome database that can be realistically used in a metagenomic analysis with typical computing facilities is circumscribed, limiting the increase of resolution beyond subspecies levels. Balancing alignment accuracy and database content is therefore an important consideration in designing the best analytical strategy. The clustering-free algorithm we previously designed and used in the WoL database to maximize the covered biodiversity given a fixed number of genomes (18) may be beneficial in this situation, but its efficacy may require further testing in the context of particular biospecimens and biological questions. Leaderboard sequencing may also be a useful strategy for iteratively augmenting the reference database with the common genomes in each sample (54). Efforts to improve algorithms, increase database coverage, and improve computing efficiency are all needed to drive advances in the field of metagenomics, and the OGU method provides an important step forward in that direction.

## MATERIALS AND METHODS

### Protocol details.

The OGU method is flexible to the type of sequence alignment. The recommended protocol, which is also the protocol demonstrated and benchmarked in this article, is as follows. Shotgun metagenomic sequencing data were aligned against the WoL reference genome database using SHOGUN v1.0.8 (42), with Bowtie2 v2.4.1 (55) as the back end. This process is equivalent to a Bowtie2 run with the following parameters: −very −sensitive −k16 −np1 −mp “1,1” −rdg “0,1” −rfg “0,1” −score −min “L,0,−0.05.”

The sequence alignment is treated as a mapping from queries (sequencing data) to subjects (reference genomes). It is possible that one sequence is mapped to multiple genomes (up to 16 using the aforementioned Bowtie2 command). In this scenario, each genome is counted 1/*k* times (*k* is the number of genomes to which this sequence is mapped). The frequencies of individual genomes were summed after the entire alignment was processed and were rounded to the nearest even integer. Therefore, the sum of OGU frequencies per sample is nearly (considering rounding) equal to the number of aligned sequences in the data set. In the output feature table, columns contain sample IDs, rows contain feature IDs (OGUs), and cell values are the frequency of each OGU in each sample. This table is ready to be analyzed using software packages such as QIIME 2 (11).

It should be noted that the Bowtie2 parameter “−k16” is part of the default protocol implemented in SHOGUN, and it was determined through benchmarks in a previous work (29). We adopted the SHOGUN protocol, but we performed benchmarks in this work to evaluate whether this parameter has a significant impact on the results (Fig. 6A and B). We also benchmarked alternative alignment methods in the framework of the OGU method (Fig. 6I to L).

It should also be noted that the OGU frequencies in the resulting table represent sequence abundance, instead of taxonomic abundance, the latter of which can be estimated by normalizing the frequencies with the sizes of the reference genomes. In Woltka, we implemented the parameter “–sizes” to enable this optional normalization. The difference between the two abundance metrics and how it impacts analysis results are discussed in reference 44.

### Implementation.

The OGU method is implemented in the bioinformatics tool Woltka (Web of Life Toolkit App), under the BSD-3-Clause open-source license. The program is written in Python 3, following high-quality software engineering standards. Its unit test coverage is 100%. The source code is hosted in the GitHub repository (https://github.com/qiyunzhu/woltka), together with instructions, tutorials, command-line references, and test data sets. The program has been included in the Python Package Index (PyPI). In addition to the standalone Woltka program, a QIIME 2 (11) plug-in is included in the software package.

Woltka automatically recognizes and parses multiplexed or per-sample sequence alignment files, either original or compressed using Gzip, Bzip2, or LZMA algorithms. It supports three alignment file formats: (i) SAM (Sequence Alignment Map) (56), which is supported by multiple short read alignment programs, such as Bowtie2 (55), BWA (57) and Minimap2 (47); (ii) the standard BLAST (58) tabular output format (-outfmt 6), which is supported by multiple-sequence-alignment programs, such as BLAST, VSEARCH (59), and DIAMOND (60); and (iii) a plain mapping of query sequences to subject genomes, which is customizable to adopt other tools and pipelines.

In addition to OGU table generation, Woltka supports summarizing features into higher-level groups. This enables taxonomic classification, for comparison purposes. The output of Woltka’s classification function and that of SHOGUN’s “assign_taxonomy” function are identical. Woltka supports three formats of classification systems: (i) the Greengenes-style lineage strings (supported by programs such as QIIME 2 [11], MetaPhlAn [43], and GTDB-tk [61]), (ii) the NCBI-style taxonomy database (62) (also known as “taxdump,” supported by programs such as Kraken 2 [5], Centrifuge [4], and DIAMOND [60]), and (iii) one or multiple plain mappings of child-to-parent classification units.

The functions implemented in Woltka that are relevant to the current work are summarized in Fig. 8.

### Deployment.

The Woltka program has been incorporated in the Qiita web analysis platform (https://qiita.ucsd.edu/) (23), as part of the standard operating procedure for analyzing shotgun metagenomic data (qp-woltka, code hosted at https://github.com/qiita-spots/qp-woltka). It can be directly launched from the graphic user interface. A job array system is used to parallelize analyses on a per-sample base to maximize processing speed. Each process uses eight cores of an Intel E5-2640 v3 CPU and 90 GB DDR4 memory. Two reference genome databases are available for user choice: (i) the WoL database (18), with 10,575 bacterial and archaeal genomes that were evenly sampled through an algorithm, and (ii) the reference and representative genomes of microbes defined in NCBI RefSeq release 200 (16). The subsequent community ecology analyses based on the OGU table are also available from Qiita. The WoL reference phylogeny is available for choice for phylogenetic analyses (such as UniFrac [63]).

This system allowed us to reanalyze all metagenomic data sets hosted on Qiita (totaling 143 studies and 57,063 samples, as of 3 March 2021) to generate OGU tables as well as tables at multiple taxonomic ranks, which are ready for subsequent meta-analysis by Qiita users. Although run time varies by sample size, the average wall clock time for analyzing one metagenomic sample (including sequence alignment against WoL using Bowtie2 and feature table generation using Woltka) was 13.8 min in this large effort.

### The HMP data set.

The Human Microbiome Project (HMP) (28) data set was downloaded from the official website (https://www.hmpdacc.org/hmp/). It contains 241 samples of 100-bp paired-end whole-genome sequencing (WGS) reads. The sequencing data had already been processed to remove human contamination and low-quality regions. We dropped samples with fewer than 1 million paired-end reads and one sample that received zero matches by SHOGUN and MIDAS (SRS057290), leaving 210 samples (Table S2). They were randomly subsampled to 1 million paired-end reads per sample. These samples represent both male (*n *=* *138) and female (*n *=* *72) human subjects. They represent seven body sites: stool (*n *=* *78), tongue dorsum (*n *=* *42), supragingival plaque (*n *=* *33), buccal mucosa (*n *=* *28), retroauricular crease (*n *=* *13), posterior fornix (*n *=* *10), and anterior nares (*n *=* *6).

10.1128/msystems.00167-22.10 TABLE S2 Metadata of the 210 HMP samples analyzed in this study. Download Table S2, XLSX file, 0.01 MB. Copyright © 2022 Zhu et al. 2022 Zhu et al. https://creativecommons.org/licenses/by/4.0/ This content is distributed under the terms of the Creative Commons Attribution 4.0 International license.

### Taxonomic profilers.

In comparison with the OGU method, we performed taxonomic profiling on the shotgun metagenomic data using five existing methods, representing two mainstream strategies for metagenome profiling and two types of output metrics (44), specified below. The default parameters were used for all programs. To maximize comparability, we used the WoL reference genome database (18) for all methods, except for MetaPhlAn (because it uses a special marker gene database which is difficult to customize).

Three profilers that utilize the information of whole genomes (sequences or *k*-mer profiles), and report sequence abundances (number of query sequences assigned to each taxon) are as follows: (i) SHOGUN v1.0.8 (42), which calls Bowtie2 v2.4.1 (56) to perform sequence alignment (note that these sequence alignments were also used for generating the OGU tables using Woltka); (ii) Bracken v2.5 (30) on the results of Kraken v2.0.8 (5); and (iii) Centrifuge v1.0.3 (4).

Two profilers that use certain sets of phylogenetically informative marker genes and report taxonomic abundances (number of genomes assigned to each taxon) were also used: (i) MetaPhlAn v2.6.0 (43) with its database (mpa_v20_m200) and (ii) MIDAS v1.3.0 (10) with the “species” command. The outputs are relative abundances and they were normalized to counts per million sequences in this study.

### Reference genome databases.

The applicability of the OGU method was tested with six reference databases of microbial genomes and the corresponding reference phylogenetic trees built by the developers. They represent a gradient of capacities, and a variety of focuses. These databases are (from smallest to largest) GEBA-I (45), UHGG (Unified Human Gastrointestinal Genome) (17), MIDAS (Metagenomic Intra-species Diversity Analysis System) (10), WoL (Web of Life) (10,575 genomes, 30.61 Gbp; developed by us and used in the default OGU protocol), GTDB89 (Genome Taxonomy Database release 89) (44) (24,771 genomes representing species clusters, 82.85 Gbp), and GEM (Genomes from Earth’s Microbiomes) (46) (OTU sequences, 45,599 genomes representing species-level OTUs, 134.82 Gbp).

### DNA sequence aligners.

The OGU method was tested using sequence alignments generated by two alternative methods, in addition to SHOGUN (which uses a specific parameter set of Bowtie2, as detailed above). They are Bowtie2 v2.4.1 (55), with its default parameter setting, and Minimap2 v2.20 (47), with its default parameter setting under the “short-read” (sr) mode.

### Beta diversity analysis.

Beta diversity analysis of the HMP data set was performed using QIIME 2 (11), following recommended protocols (64). Specifically, beta diversity distance matrices were constructed using the “qiime diversity beta” command with Jaccard and Bray-Curtis metrics and using the “qiime diversity beta-phylogenetic” command (65) with unweighted UniFrac and weighted UniFrac metrics, based on the WoL reference phylogeny. PCoA was performed using the “qiime diversity pcoa” command. The correlation between biological factors (body site and host sex) and beta diversity was assessed using PERMANOVA, through the command “qiime diversity adonis,” with 999 permutations (the default setting).

### Site clustering by environment.

In the HMP study, we quantified the proximity of the three oral sites (tongue dorsum, supragingival plaque, and buccal mucosa) compared with the four nonoral sites (stool, retroauricular crease, posterior fornix, and anterior nares) as follows: For each sample in the three oral sites, we calculated the beta diversity distance to all samples in all but the current site. We then separated these distances into oral (i.e., the two oral sites other than the current one) and nonoral (i.e., the four nonoral sites). We calculated the ratio of the mean distance of the former versus the latter. Finally, we reported the distribution of the mean ratios of all oral samples.

### Phylogenetic factorization.

We performed phylogenetic factorization as implemented in Phylofactor v0.0.1 to infer phylogenetic clades (“factors”) that are differentially abundant between male and female subjects. Two samples with fewer than 100,000 OGU counts were excluded from the analysis. OGUs with relative abundance below 0.01% were dropped from each sample, and OGUs present in fewer than two samples were also excluded. We built an explained variance-maximizing (the choice parameter was set to “var”) Phylofactor model using the OGU table and the WoL phylogeny. We specified the model to return 20 factors. They were labeled by the taxonomic annotation of the corresponding phylogenetic clades as provided in the WoL database. The results were visualized with EMPress. In each factor, we tested the differences in male versus female subjects by comparing the isometric log-ratio-transformed vectors corresponding to each sample group using a two-tailed independent-sample *t* test.

### The FINRISK 2002 data set.

The FINRISK 2002 is a large, well-phenotyped, and representative cohort based on a stratified random sample of the population aged 25 to 74 years from specific geographical areas of Finland (36). All volunteer participants took a self-administered questionnaire, physical measurements, and collection of blood and stool samples. The microbiome data and metadata that support the findings of this study are available from the THL Biobank based on a written application and following relevant Finnish legislation. Details of the application process are described on the Biobank website (https://thl.fi/en/web/thl-biobank/for-researchers).

Paired 16S rRNA gene amplicon sequencing data and shotgun metagenomic sequencing data are available for 6,430 stool samples. The 16S rRNA data were demultiplexed, quality filtered, and denoised with deblur v1.1.0 (66), resulting in an average ASV frequency of 8,787 per sample. Taxonomic classification was performed using a pretrained naive Bayes classifier against the Greengenes 13_8 database at an OTU clustering level of 99%. Feature tables were rarefied to a sampling depth of 10,000. The shotgun metagenomic data were trimmed and quality filtered using Atropos v1.1.25 (67), resulting in an average of 1.07 million paired-end sequences per sample. They were aligned to the WoL database using SHOGUN v1.0.8. An OGU table was generated using the current approach. As a comparison, Bracken v2.5 with Kraken v2.0.8 was used to infer taxonomic profiles using the same WoL database. These analyses were the same as the corresponding analyses of the HMP shotgun metagenomic data set, as described above.

### Supervised regression for age prediction.

We performed machine learning analysis of microbial profiles derived from both 16S amplicon sequencing and shotgun metagenomics sequencing, at distinct levels of resolution. These included taxonomic ranks (phylum, class, order, family, genus, and species) for both 16S rRNA and shotgun metagenomic data (the latter of which were inferred by either SHOGUN or Bracken), ASVs for 16S rRNA data, and OGU for shotgun metagenomic data (inferred by SHOGUN with Woltka). In each profile, features with a study-wide prevalence less than 0.001 were excluded. Random forest regressors for predicting chronological age were trained based on each profile with tuned hyperparameters with a stratified 5-fold cross-validation approach using the R package ranger v0.12.1 (68). Each data set was split into five groups with similar age distributions, and we trained the classifier on 80% of the data and made predictions on the remaining 20% of the data in each fold iteration. We next evaluated the performance of age prediction using mean absolute error (MAE), calculated as

$$\frac{\sum_{i=1}^{n}\left|y_{i}\;-\;x_{i}\right|}{n}$$

where *y* denotes the predicted age, *x* denotes the chronological age, and *n* is the total number of samples. Based on the MAE evaluation, we next determined the most predictive taxonomic levels derived from both 16S and shotgun metagenomics.

To identify the most important taxonomic features that contributed to the age prediction, we visualized the 128 top-ranked important features by built-in random forest importance scores and their phylogenetic relationships using EMPress (33). We next performed the feature selection analysis to identify a set of important microbial features that can maximize the model performance. We built age regressors using a series of reduced sets (*n* = 2, 4, 8, 16, 32, 64, 128, 256, 512, 1,024, and the number of all features) of the most predictive taxonomic features (namely, OGU) and compared their performance. The rationale was to observe an optimized MAE while adding features into the regression model.

### Simulated data set and assignment accuracy.

We evaluated the assignment accuracy of OGUs and species using a simulated metagenomic data set, generated following the protocol described in reference 44. Specifically, this data set has 25 samples simulated to represent the real microbial communities collected from a variety of habitats (*n *=* *5 each): built environment, human gut, mouth, skin, and vagina. We first fixed the species-level richness for each habitat and selected representative genomes for each species as previously identified in these habitats. Their abundances were created randomly from a log-normal distribution. Using the ground truth as reference, we calculated three metrics: (i) AUPR (area under the precision-recall curve), which evaluates the presence/absence of features (41); (ii) Bray-Curtis, which evaluates the abundance of features, and (iii) weighted UniFrac, which also evaluates the abundance and used the WoL phylogeny. It should be noted that the abundance evaluated here was the sequence abundance (see reference 44 for a thorough discussion). We evaluated both OGU and species profiles predicted by SHOGUN and species profiles predicted by Bracken and MIDAS. All three tools used the WoL database so that their results are comparable (see above). Strictly speaking, the results of OGUs and species are not directly comparable because they used different references (genomes or species). Nevertheless, they implicated what to expect with individual methods.

### UniFrac with a taxonomic tree.

The taxonomic hierarchies of the WoL reference genomes were converted into a Newick-formatted tree, in which the branches connecting two adjacent taxonomic ranks have a constant length. Only the seven standard tanks (kingdom, phylum, class, order, family, genus, and species) were considered. This ensures that all taxa at the same rank have the same tip-to-root length. This tree was used as a replacement of the phylogenetic tree for UniFrac analyses.

### Collapsing phylogenetic tree to taxonomic ranks.

The WoL reference phylogeny (18) was collapsed such that each tip represents a taxonomic unit at a given rank. We used the taxonomy curated during the WoL project (see reference 18 for details) so that every taxonomic unit is a monophyletic group in the phylogenetic tree, which allowed consistent conversion of the tree of genomes into trees of each of the six taxonomic ranks. The resulting trees were used in the UniFrac analyses of the taxonomic profiles.

### Generating the half-sized and double-sized genome pools.

The WoL database contains 10,575 genomes sampled from 86,200 nonredundant microbial genomes available from NCBI at the time of construction (18). They were sampled using a combination of criteria, the center of which was a “prototype selection” algorithm we developed, which ensures that the total biodiversity (measured by MinHash distance) covered by a fixed number of genomes is maximized (18). We used the same algorithm to sample half of the genomes (5,287) from the WoL genome pool. Next, we used the same algorithm to sample twice as many genomes (21,150) from the original pool of 86,200 genomes, with the WoL genomes as “seeds” (detailed in reference 18). These procedures ensured that the half-sized genome pool is a subset of the WoL pool, which in turn is a subset of the double-sized pool.

### Building reference phylogenies of the half-sized and double-sized genome pools.

The reference phylogeny of the half-sized genome pool was pruned from the original WoL reference phylogeny. For the double-sized genome pool, we started with the same sequence data that were used for building the original WoL reference phylogeny (18). They were the 381 global marker genes inferred by PhyloPhlAn and curated by the authors. Next, we adopted a relatively scalable protocol (to overcome the significant size of the genome pool) that was implemented in PhyloPhlAn 3.0 (22) to build a phylogenomic tree from the 381 genes by 21,150 genomes. Specifically, we used MAFFT v7.480 (69) to align protein sequences of each marker gene family, then used trimAl v1.4.rev15 (70) to conduct quality trimming of the multiple sequence alignments, and then used PhyloPhlAn 3.0’s built-in algorithm (corresponding to the parameters –fast and –diversity high) to further trim the alignments and to select 10% sites that carry the strongest phylogenetic signals. The processed alignments were concatenated into a master alignment of 14,583 sites. We then used the maximum-likelihood method implemented in FastTree v2.1.10 (71). The parameter settings for MAFFT, trimAl, and FastTree followed the default protocol of PhyloPhlAn 3.0.

### Subsampling of OGU tables.

To assess the impact of sequencing depth on analysis results, we randomly subsampled the OGU tables to lower depths (sum of OGU frequencies per sample). This process mimicked lower sequencing depths in the original data, because the sum of OGU frequencies is equal to the number of aligned sequences (see above). This process further considered the unaligned part of the sequencing data. For example, if *m* out of *n* sequences in a sample were aligned to at least one reference genome (therefore, the sum of OGU frequencies was *m*), we added an extra “unaligned” feature of frequency of *n* − *m* to the OGU table, prior to random subsampling, and removed this extra feature after sampling. For each sampling depth, we generated 10 replicates using random seeds 0 to 9.

### Subsampling of Bracken results.

To simulate the effect of subsampling original sequencing data, we performed random subsampling of the lines in the Kraken2 mapping files, which record the frequencies of *k*-mers that match individual taxonomic units for each query sequence. Similarly (see above), the unclassified portion was considered, and 10 replicates were generated for each depth. Bracken was then executed on the subsampled Kraken2 mapping files to generate species-level profiles.

### Subsampling of MIDAS results.

We performed random subsampling on the temporary files generated in the original MIDAS runs. These temporary files record the BLAST search results of the sequencing data against predefined marker gene sequences. Because one query sequence may match multiple targets, the subsampling process took each query sequence as the unit instead of each line of the file. Similarly (see above), the unclassified portion was considered, and 10 replicates were generated for each depth. MIDAS was then executed on the subsampled BLAST search results to generate species-level profiles.

### Statistical analysis.

All data analysis was performed using QIIME 2 release 2020.6. PERMANOVA was performed using the adonis command (which wraps the adonis function in vegan v2.5-6). The *P* value was calculated using one million permutations. A paired *t* test was performed using the ttest_rel function in SciPy v1.4.1.
