# Correction for Zhu et al., “Phylogeny-Aware Analysis of Metagenome Community Ecology Based on Matched Reference Genomes while Bypassing Taxonomy”

**DOI:** 10.1128/msystems.01289-24

**Published:** 2024-10-18

**Authors:** Qiyun Zhu, Shi Huang, Antonio Gonzalez, Imran McGrath, Daniel McDonald, Niina Haiminen, George Armstrong, Yoshiki Vázquez-Baeza, Julian Yu, Justin Kuczynski, Gregory D. Sepich-Poore, Austin D. Swafford, Promi Das, Justin P. Shaffer, Franck Lejzerowicz, Pedro Belda-Ferre, Aki S. Havulinna, Guillaume Méric, Teemu Niiranen, Leo Lahti, Veikko Salomaa, Ho-Cheol Kim, Mohit Jain, Michael Inouye, Jack A. Gilbert, Rob Knight

## AUTHOR CORRECTION

Volume 7, no. 2, e00167-22, 2023, https://doi.org/10.1128/msystems.00167-22. Page 1, Notes: “The authors declare no conflict of interest” should read as follows. “Rob Knight is a scientific advisory board member and consultant for BiomeSense, Inc., has equity, and receives income. He is a scientific advisory board member and has equity in GenCirq. He is a consultant and scientific advisory board member for DayTwo and receives income. He has equity in and acts as a consultant for Cybele. He is a co-founder of Biota, Inc., and has equity. He is a cofounder of Micronoma, has equity, and is a scientific advisory board member. The terms of these arrangements have been reviewed and approved by the University of California, San Diego, in accordance with its conflict-of-interest policies. Jack A. Gilbert is a cofounder, has equity, and is a Scientific Advisory Board member for BiomeSense, Inc. The terms of this arrangement have been reviewed and approved by the University of California, San Diego, in accordance with its conflict-of-interest policies. Daniel McDonald is a consultant for BiomeSense, Inc., has equity, and receives income. The terms of these arrangements have been reviewed and approved by the University of California, San Diego, in accordance with its conflict-of-interest policies."

This correction does not change the results or the conclusions of this work.

