# Supplementary figures and images for: Phylogeny-Aware Analysis of Metagenome Community Ecology Based on Matched Reference Genomes while Bypassing Taxonomy
Source: mSystems. 2022 Apr 4;7(2):e00167-22. doi: 10.1128/msystems.00167-22 (PMC9040630; doi:10.1128/msystems.00167-22)

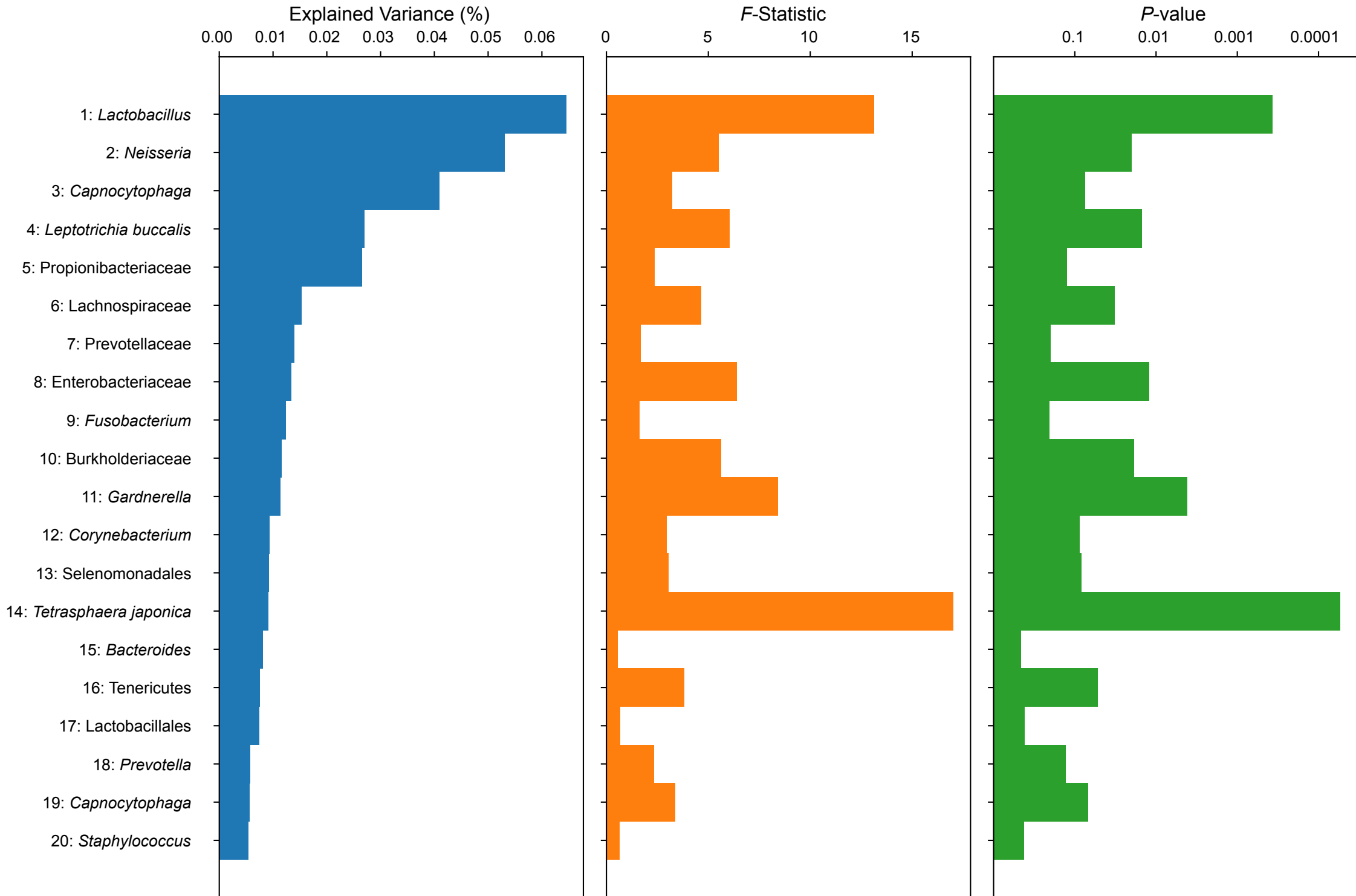

Supplement: FIG S1 [file msystems.00167-22-sf001.pdf]

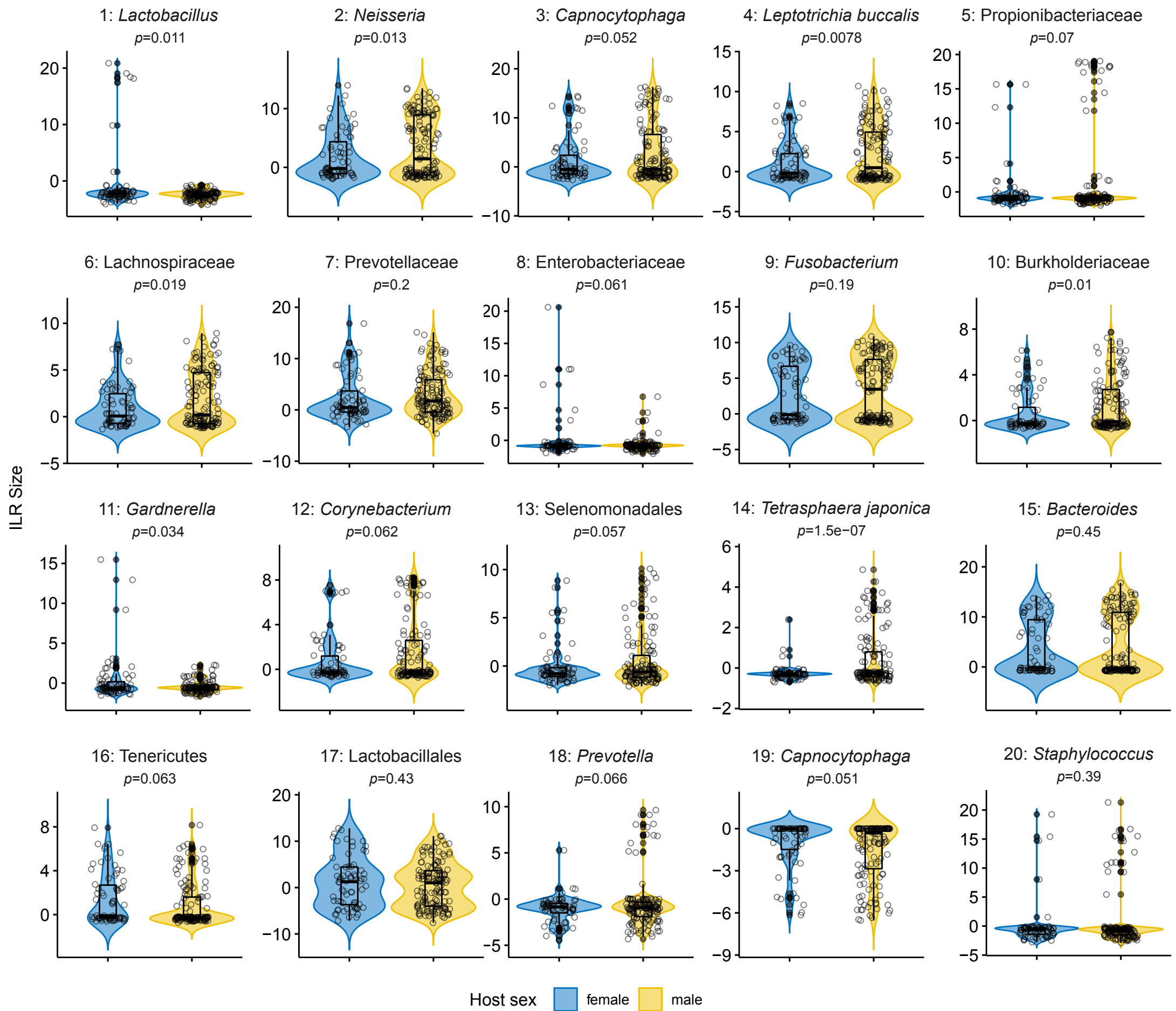

Supplement: FIG S2 [file msystems.00167-22-sf002.pdf]

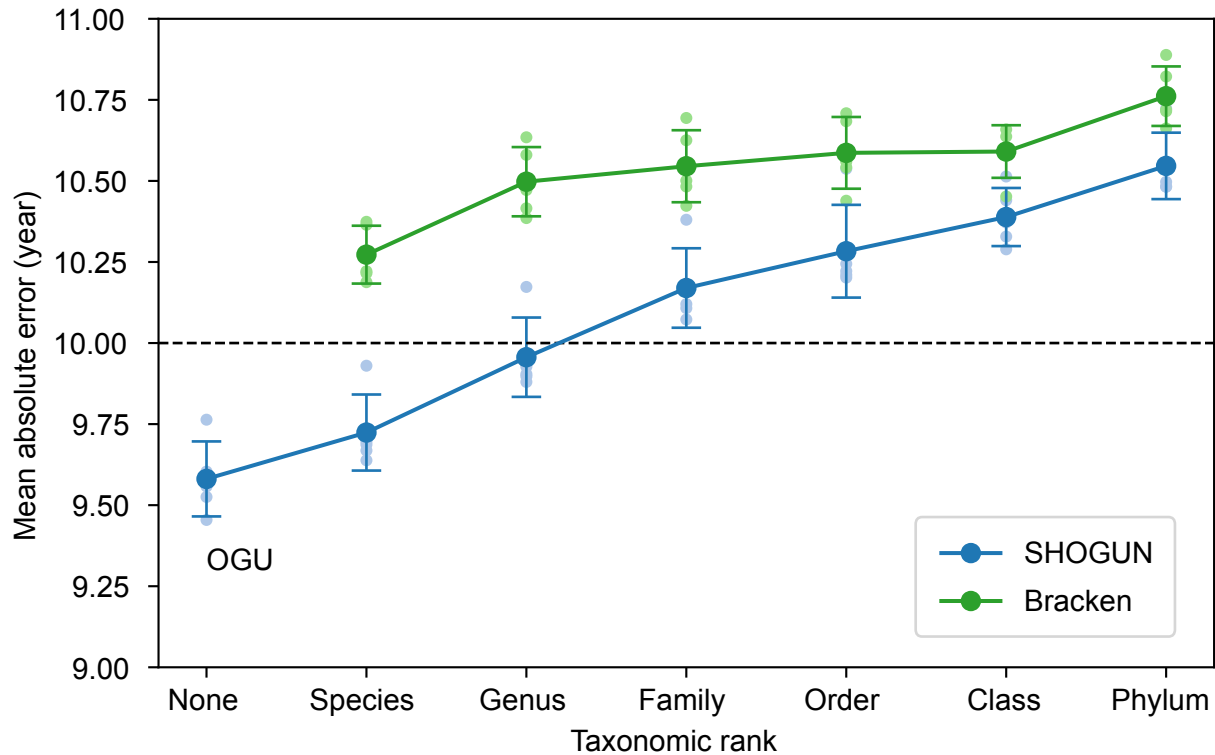

Supplement: FIG S3 [file msystems.00167-22-sf003.pdf]

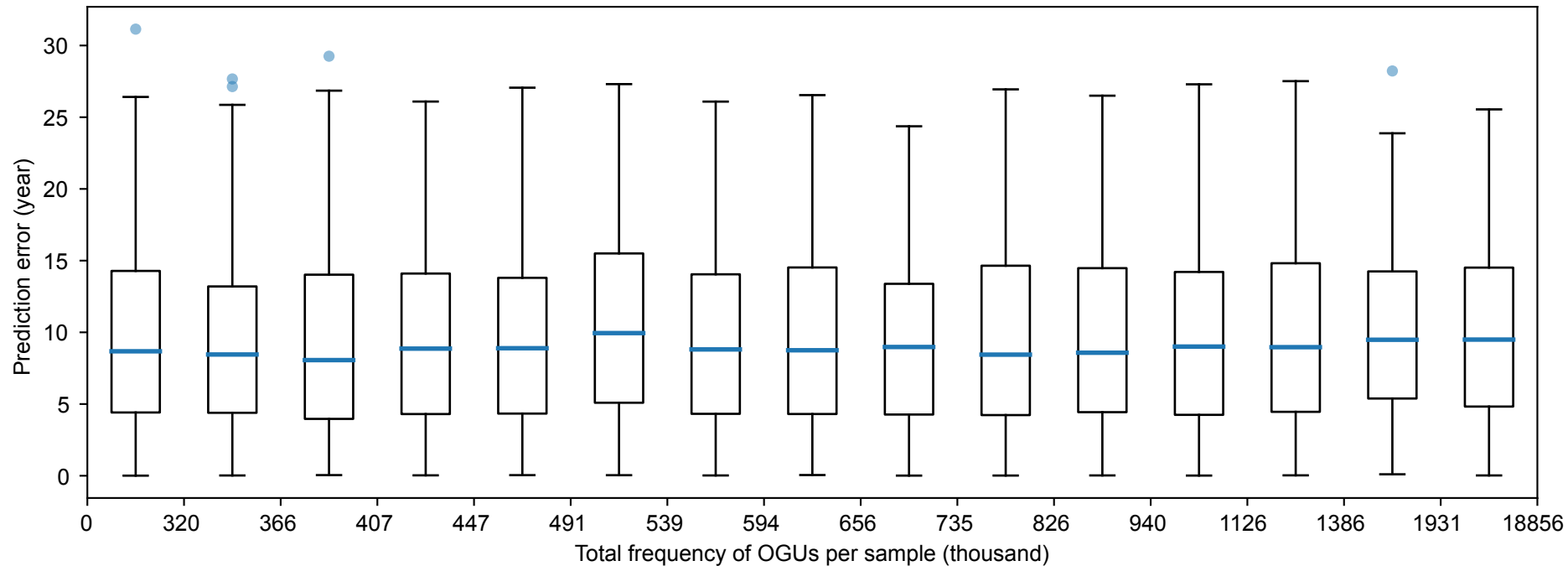

Supplement: FIG S4 [file msystems.00167-22-sf004.pdf]

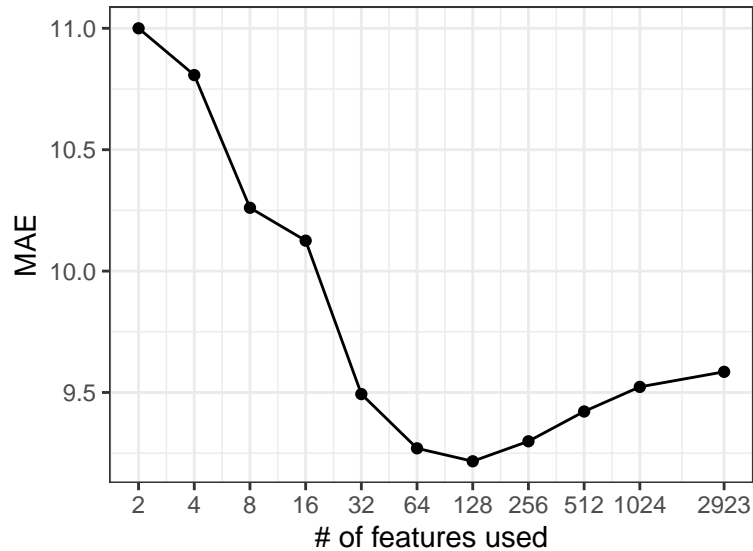

Supplement: FIG S5 [file msystems.00167-22-sf005.pdf]

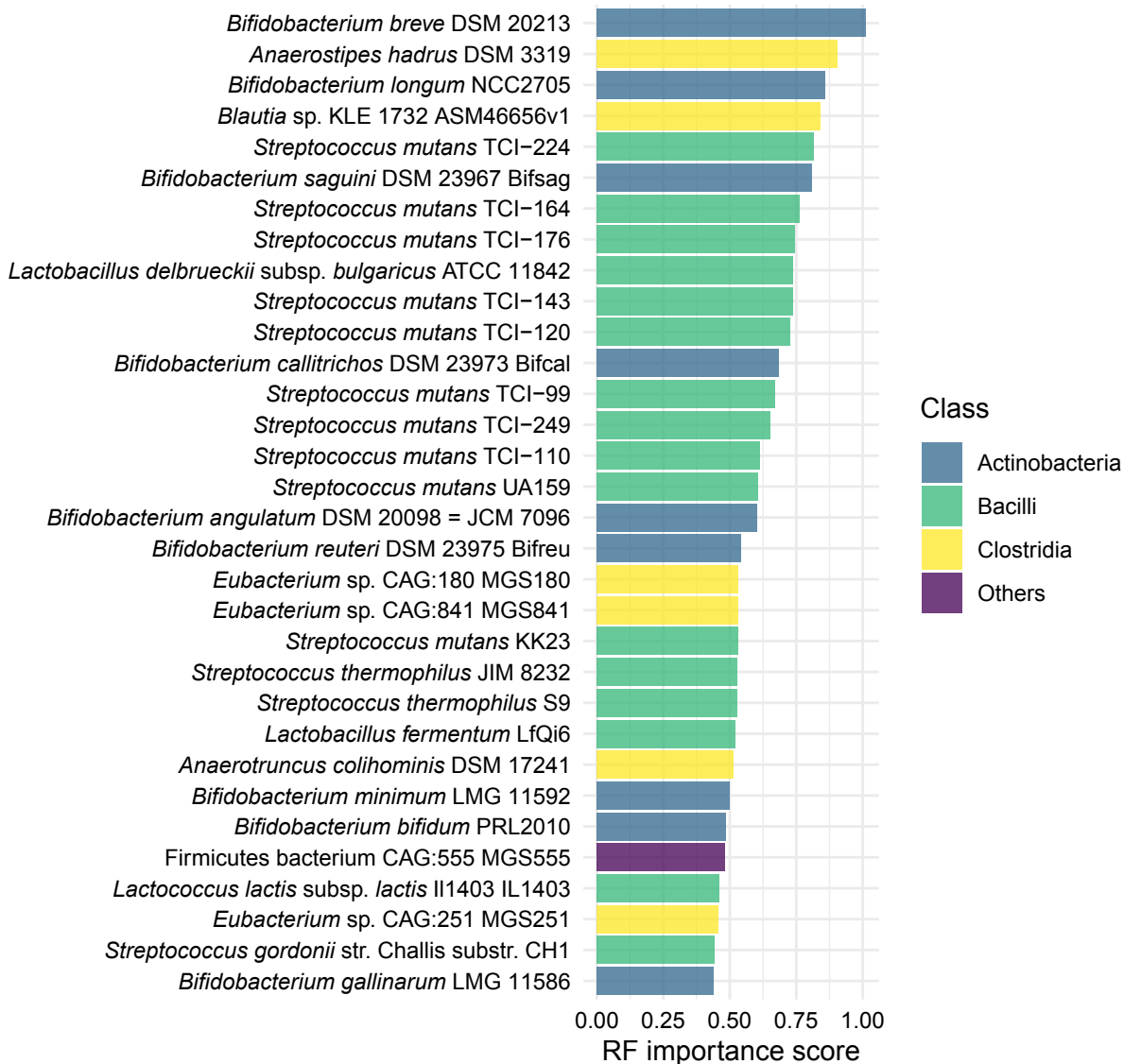

Supplement: FIG S6 [file msystems.00167-22-sf006.pdf]

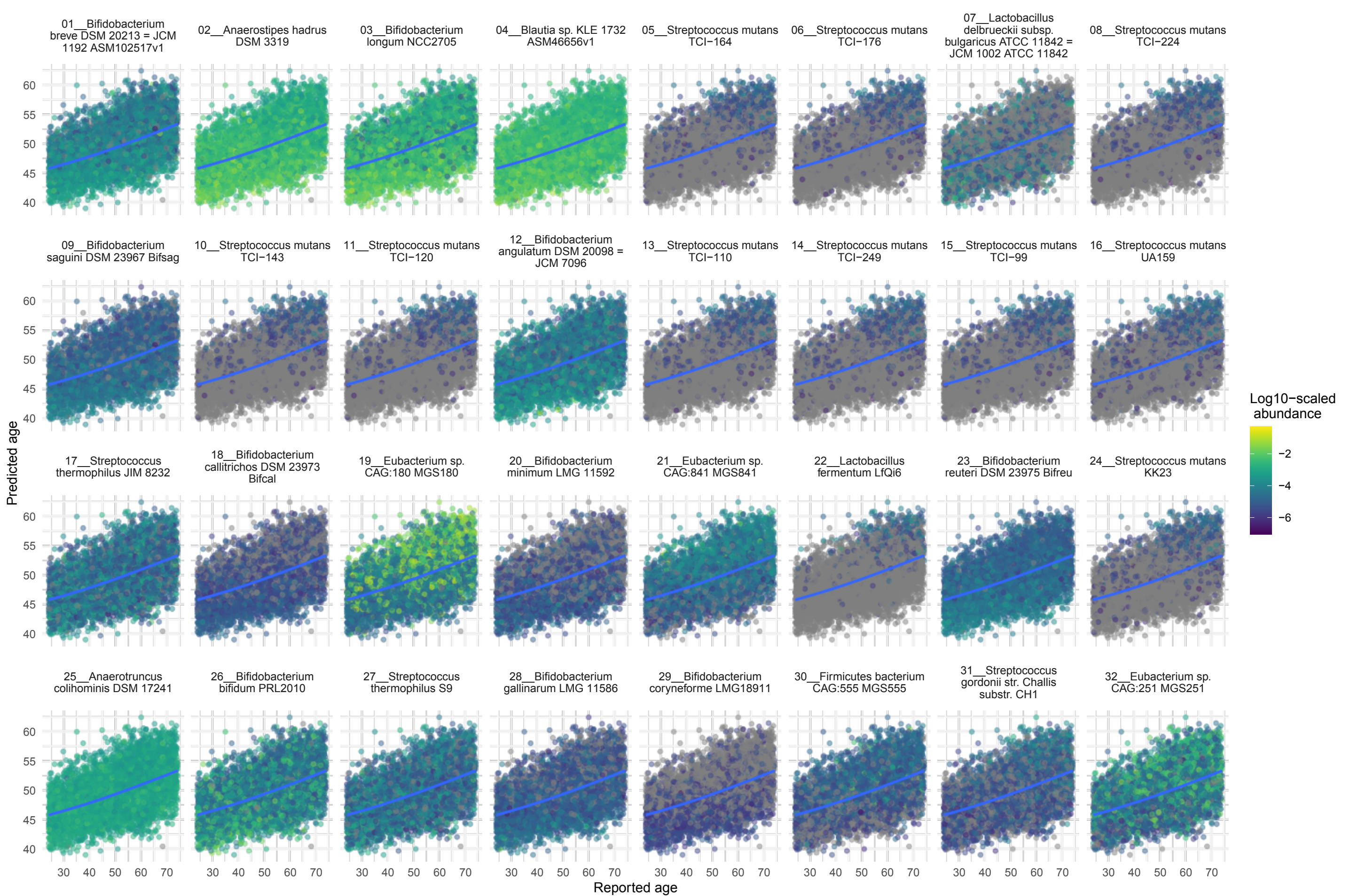

Supplement: FIG S7 [file msystems.00167-22-sf007.pdf]
