# Supplementary material for: Phylogeny-Aware Analysis of Metagenome Community Ecology Based on Matched Reference Genomes while Bypassing Taxonomy
Source: mSystems. 2022 Apr 4;7(2):e00167-22. doi: 10.1128/msystems.00167-22 (PMC9040630; doi:10.1128/msystems.00167-22)
Supplement: FIG S8 [file msystems.00167-22-sf008.pdf]

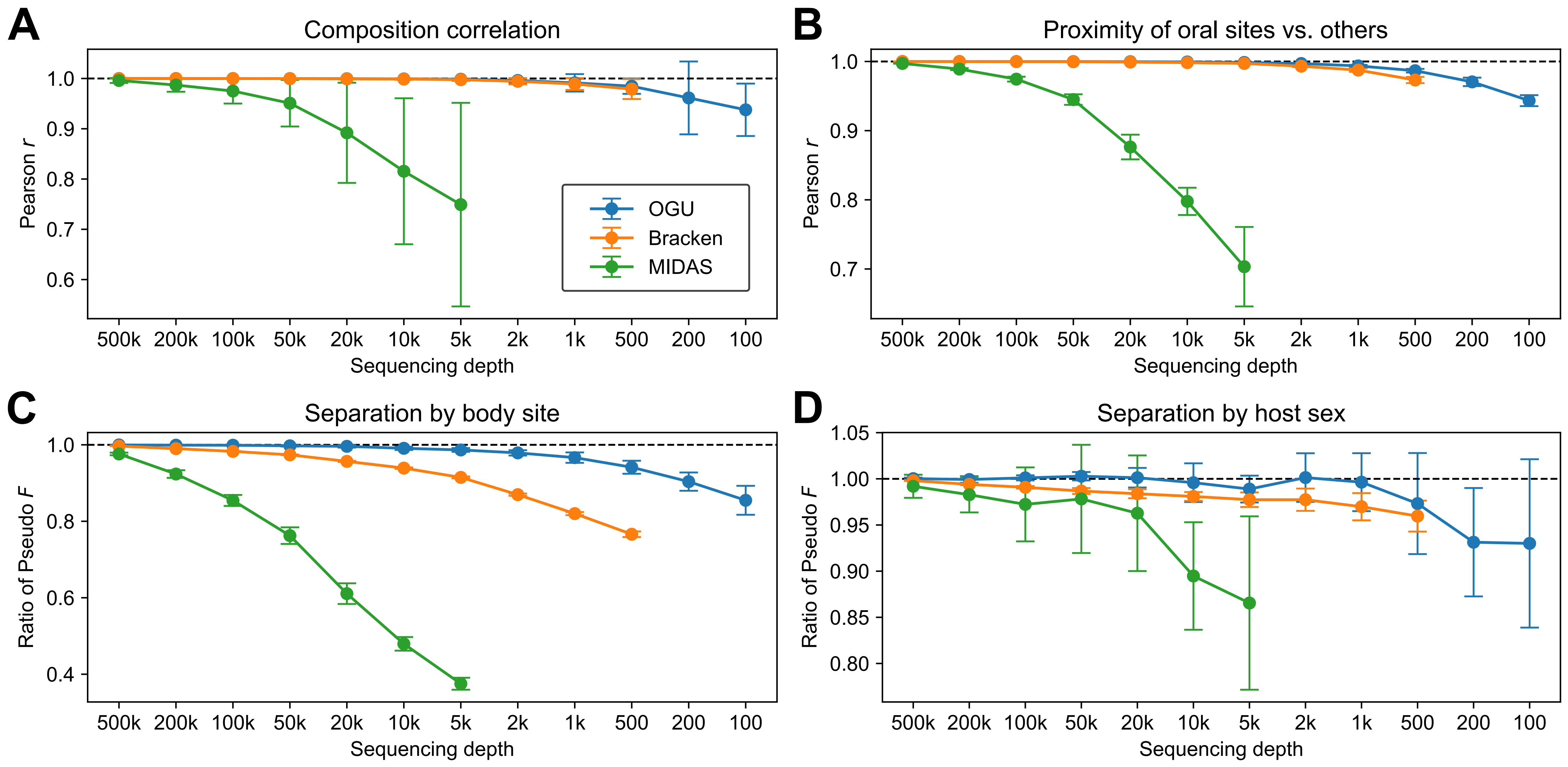

A
Composition correlation
Pearson r
1.0
0.9
0.8
0.7
0.6
OGU
Bracken
MIDAS
500k 200k 100k 50k 20k 10k 5k 2k 1k 500 200 100
Sequencing depth
B
Proximity of oral sites vs. others
Pearson r
1.0
0.9
0.8
0.7
500k 200k 100k 50k 20k 10k 5k 2k 1k 500 200 100
Sequencing depth
C
Separation by body site
Ratio of Pseudo F
1.0
0.8
0.6
0.4
500k 200k 100k 50k 20k 10k 5k 2k 1k 500 200 100
Sequencing depth
D
Separation by host sex
Ratio of Pseudo F
1.05
1.00
0.95
0.90
0.85
0.80
500k 200k 100k 50k 20k 10k 5k 2k 1k 500 200 100
Sequencing depth
